# Supplementary material for: Deep neuromuscular block for minimally invasive lung surgery: a protocol for a systematic review with meta-analysis and trial sequential analysis
Source: BMJ Open. 2022 May 23;12(5):e056816. doi: 10.1136/bmjopen-2021-056816 (PMC9131110; doi:10.1136/bmjopen-2021-056816)
Supplement: Supplementary data [file bmjopen-2021-056816supp001.pdf]

Supplementary appendix 1: Search strategy

Search strategy of PubMed as follows:

#1 “neuromuscular blockade"[MeSH Terms] OR neuromusc*[tiab]” OR “muscle relaxation” [MeSH Terms]

#2 “Deep[tiab] OR profound[tiab] OR intense[tiab] OR extreme[tiab] OR depth[tiab]”

#3 “Pulmonary” [Mesh] OR “Lung” [Mesh] OR Pulmonary [tiab] OR Lung [tiab]

#4 “Surgical Procedures Operative” [Mesh] OR “Microsurgery” [Mesh] OR “Surgical Procedures Minimally Invasive” [Mesh] OR Minimally Invasive Surgery[tiab] OR MIS [tiab] OR Minimal Access Surgical Procedures [tiab]OR Minimal Surgical Procedures[tiab] OR Minimally Invasive Surgical Procedures [tiab] OR Minimal Surgical Procedure[tiab] OR minimally invasive surgical procedure [tiab] OR minimal access surgical procedure[tiab]

#5 “Thoracic surgery, Video-Assisted” [Mesh] or Surgeries, Video-Assisted Thoracic [af] or Surgery, Video-Assisted Thoracic [af] or Thoracic Surgeries, Video-Assisted [af] or Thoracic surgery, Video-Assisted [af] or Video-Assisted Thoracic Surgeries [af] or Video-Assisted Thoracic Surgery [af] or Surgeries, Video-Assisted Thoracoscopic [af] or Surgery, Video-Assisted Thoracoscopic [af] or Thoracoscopic Surgeries, Video-Assisted [af] or Thoracoscopic Surgery, Video-Assisted [af] or Video Assisted Thoracoscopic Surgery [af] or Video Assisted Thoracoscopic Surgeries [af] or Video-Assisted Thoracic Surgery [af] or Video Assisted Thoracic Surgery [af] or Surgery, Thoracic, Video-Assisted [af] or VATS [af] or VATSs [af].

#6 “Robotics” [MeSH] OR robot* [tiab] OR computer guid*[tiab] OR computer-guid*[tiab] OR computer-assisted[tiab] OR computer assisted [tiab]OR da Vinci [tiab]OR Zeus [tiab]OR telesurgery[tiab]

#7 #1 AND #2 AND #3

#8 #4 OR #5 OR #6

#9 “controlled clinical trial” [Publication Type] OR “randomized controlled trial” [Publication Type] OR “randomized” [Title/Abstract] OR “randomized” [Title/Abstract] OR “Placebo” [Title/Abstract] OR “randomly” [Title/Abstract] OR “Clinical trial” [Title]

#10 (animals [MeSH Terms]) NOT ((human [MeSH Terms]) AND (animals [MeSH Terms]))

#11 #7and #8 and #9 not #10

Search strategy of Cochrane library as follows:

#1 MeSH descriptor: [neuromuscular blockade] explode all trees

#2 MeSH descriptor: [muscle relaxation] explode all trees

#3 (neuromusc*): ti,ab,kw

#4 #1 or # 2 or # 3

#5 (Deep): ti,ab,kw or (profound):ti,ab,kw or (intense):ti,ab,kw or (extreme):ti, ab,kw or (depth):ti,ab,kw

#6 #4 and #5

#7 MeSH descriptor: [Pulmonary] explode all trees

#8 MeSH descriptor: [Lung] explode all trees

#9 #7 or # 8

#10 #6 and #9

#11 MeSH descriptor: [Surgical Procedures Operative] explode all trees

#12 MeSH descriptor: [Thoracic surgery, Video-Assisted] explode all trees

#13 MeSH descriptor: [Microsurgery] explode all trees

#14 MeSH descriptor: [Surgical Procedures Minimally Invasive] explode all trees

#15 MeSH descriptor: [Robotics] explode all trees

#16 (surgery or surgical* or Video-Assisted* or Video Assisted* or Video* or Thorac* or VATS): ti,ab,kw

#17 (robot*): ti,ab,kw

#18 (computer guid* OR computer-guid* OR computer-assisted OR computer assisted): ti,ab,kw

#19 (da Vinci OR Zeus OR telesurgery): ti,ab,kw

#20 #11 or # 12 or #13 or # 14 or #15 or # 16 or #17 or # 18 or #19

#21 #10 and # 20

#22 (controlled clinical trial):pt or (randomized controlled trial):pt or (random*): ti,ab,kw or (Clinical trial):ti,ab,kw

#23 #21 and #22

Search strategy of Web of Science as follows:

#1 TS= (neuromuscular blockade or neuromusc* or muscle relaxation)

#2 TS= (Deep or profound or intense or extreme or depth)

#3 TS= (Pulmonary or Lung)

#4 TS= (Surgical Procedures Operative or Thoracic surgery, Video-Assisted or Microsurgery or Surgical Procedures Minimally Invasive or Robotics)

#5TS= (surgery or surgical* or Video-Assisted* or Video Assisted* or Video* or Thorac* or VATS or robot* or computer guid* or computer-guid* or computer-assisted or computer assisted or da Vinci or Zeus or telesurgery)

#6 #4 OR #5

#7 #1 and #2 and #3 and #6

#8 TS= (random* or Clinical trial)

#9 #7 and #8

Search strategy for Ovid Medline as follows:

#1 exp neuromuscular blockade/ or exp muscle relaxation/ or neuromusc*.mp.

#2 (Deep or profound or intense or extreme or depth) .mp.

#3 exp pulmonary / or exp Lung

#4 #1 and #2 and #3

#5 exp surgical procedures operative/ or exp Thoracic surgery, Video-Assisted / or exp Microsurgery/or exp Surgical Procedures Minimally Invasive/ or exp Robotics/

#6 (surgery or surgical*).mp.

#7 (Video-Assisted* or Video Assisted* or Video* or Thorac* or VATS).mp.

#8 (robot*).mp.

#9(computer guid* or computer-guid* or computer-assisted or computer assisted).mp.

#10(da Vinci or Zeus or telesurgery).mp.

#11 #5or #6 or #7 or #8 or #9 or #10

#12 randomized controlled trial.pt.

#13 controlled clinical trial.pt.

#14 randomized.ab.

#15 placebo.ab.

#16 clinical trials as topic.sh.

#17 randomly.ab.

#18 trial.ti.

#19 #12 or #13 or #14 or #15 or #16 or #17 or #18

#20 (animals not (humans and animals)).sh.

#21 #19 not #20

#22 #4 and #11 and #21

Search strategy for Embase as follows:

#1 exp neuromuscular blockade/

#2 neuromusc*.mp.

#3 exp muscle relaxation/

#4 (Deep or profound or intense or extreme or depth).mp.

#5 exp Pulmonary /

#6 exp Lung/

#7 exp Surgical Procedures Operative/

#8 exp Thoracic surgery, Video-Assisted /

#9 exp Microsurgery/

#10 exp Surgical Procedures Minimally Invasive/

#11 exp Robotic/

#12 (surgery or surgical*).mp.

#13 (robot*).mp.

#14(Video-Assisted* or Video Assisted* or Video* or Thorac* or VATS).mp.

#15 (computer guid* or computer-guid* or computer-assisted or computer assisted).mp.

#16 (da Vinci or Zeus or telesurgery).mp.

#17 Clinical trial.mp.

#18 (placebo*).mp.

#19 exp randomized controlled trial/

#20 (random*).mp.

#21 (exp animal/ or nonhuman/ or exp animal experiment/) not human/

#22 #1 or #2 or #3

#23 #22 and #4

#24 #5 or #6

#25 #7 or #8 or #9 or #10 or #11 or #12 or #13 or #14 or #15 or #16

#26 #23 and #24 and #25

#27 #17 or #18 or #19 or #20

#28 #26 and #27

#29 #28 not #21

WHO ICTRP Trial registry

http://apps.who.int/trialsearch (WHO ICTRP register) will be searched via the advanced search page. Search terms were: (Lung or Pulmonary) AND (minimal invasive or minimally invasive or surgery or surgical procedures operative or microsurgery or Video-Assisted* or Video Assisted* or Video* or Thorac* or VATS or robot* or computer guid* or computer-guid* or computer-assisted or computer assisted or da Vinci or Zeus or telesurgery) AND (Deep neuromuscular blockade or profound neuromuscular blockade or intense neuromuscular blockade or extreme neuromuscular blockade or depth neuromuscular blockade).

Clinicaltrials.gov search strategy

http://clinicaltrials.gov (NIH register) will be searched via advanced search page. Search terms were: Condition or disease: (Lung or Pulmonary) AND (minimal invasive or minimally invasive or surgery or surgical procedures operative or microsurgery or Video-Assisted* or Video Assisted* or Video* or Thorac* or VATS or robot* or computer guid* or computer-guid* or computer-assisted or computer assisted or da Vinci or Zeus or telesurgery).

Study type: Interventional Studies.

Intervention/treatment: (Deep neuromuscular blockade or profound neuromuscular blockade or intense neuromuscular blockade or extreme neuromuscular blockade or depth neuromuscular blockade)

Chinese database

China National Knowledge Infrastructure (CNKI) search strategy

(电视胸腔镜 or 胸腔镜 or 腔镜 or 微创 or 机器人 or 机器人辅助 or 达芬奇 or 宙斯 or RATS or VATS or 多孔 VATS or 四孔 VATS or 三孔 VATS or 两孔 VATS or 单孔 VATS) and (手术 or 切除术 or 根治术) and (肺 or 肺癌 or 肺肿瘤) and (深度肌松 or 深肌松 or 深度神经肌肉阻滞)and (随机 or 对照)

Chinese BioMedical Literature (CBM)

("电视胸腔镜" [全部字段] or"胸腔镜" [全部字段] or "腔镜" [全部字段] or "微创" [全部字段]

or "机器人" [全部字段] or "机器人辅助" [全部字段] or "达芬奇" [全部字段] or "宙斯" [全部字段] or " RATS " [全部字段] or "VATS [全部字段] or "多孔 VATS" [全部字段]or "四孔 VATS " [全部字段] or "三孔 VATS" [全部字段] or "两孔 VATS" [全部字段] or "单孔 VATS" [全部字段]) and ("手术" [全部字段] or "切除术" [全部字段] or"根治术" [全部字段]) and ("肺" [全部字段] or "肺癌" [全部字段] or"肺肿瘤" [全部字段]) and ("深度肌松" [全部字段] or "深肌松" [全部字段] or"深度神经肌肉阻滞" [全部字段]) and ("随机" [全部字段] or "对照" [全部字段])

VIP database

关键词=(电视胸腔镜 or 胸腔镜 or 腔镜 or 微创 or 机器人 or 机器人辅助 or 达芬奇 or 宙斯 or RATS or VATS or 多孔 VATS or 四孔 VATS or 三孔 VATS or 两孔 VATS or 单孔 VATS) AND 关键词= (手术 or 切除术 or 根治术) AND 关键词= (肺 or 肺癌 or 肺肿瘤) AND 关键词= (深度肌松 or 深肌松 or 深度神经肌肉阻滞)AND 关键词= (随机 or 对照)

Wan fang database.

(电视胸腔镜 or 胸腔镜 or 腔镜 or 微创 or 机器人 or 机器人辅助 or 达芬奇 or 宙斯 or RATS or VATS or 多孔 VATS or 四孔 VATS or 三孔 VATS or 两孔 VATS or 单孔 VATS) and (手术 or 切除术 or 根治术) and (肺 or 肺癌 or 肺肿瘤) and (深度肌松 or 深肌松 or 深度神经肌肉阻滞)and (随机 or 对照)
