# Supplementary material for: Deep neuromuscular block for minimally invasive lung surgery: a protocol for a systematic review with meta-analysis and trial sequential analysis
Source: BMJ Open. 2022 May 23;12(5):e056816. doi: 10.1136/bmjopen-2021-056816 (PMC9131110; doi:10.1136/bmjopen-2021-056816)
Supplement: Supplementary data [file bmjopen-2021-056816supp002.pdf]

Assessment of risk of bias in included studies

Random sequence generation

- ***Low risk:*** If sequence generation was achieved using computer random number generator or a random number table. Drawing lots, tossing a coin, shuffling cards, and throwing dice were also considered adequate if performed by an independent adjudicator.
- ***Unclear risk:*** If the method of randomisation was not specified, but the trial was still presented as being randomised.
- ***High risk:*** If the allocation sequence is not randomised or only quasi-randomised. These trials will be excluded.

Allocation concealment

- ***Low risk:*** If the allocation of patients was performed by a central independent unit, onsite locked computer or identical-looking numbered sealed envelopes.
- ***Uncertain risk:*** If the trial was classified as randomised but the allocation concealment process was not described.
- ***High risk:*** If the allocation sequence was familiar to the investigators who assigned participants.

Blinding of participants and treatment providers

- ***Low risk:*** If the participants and the treatment providers were blinded to intervention allocation and this was described.
- ***Uncertain risk:*** If the procedure of blinding was insufficiently described.
- ***High risk:*** If blinding of participants and the treatment providers was not performed.

Blinding of outcome assessment

- ***Low risk of bias:*** If it was mentioned that outcome assessors were blinded and this was described.
- ***Uncertain risk of bias:*** If it was not mentioned if the outcome assessors in the trial were blinded or the extent of blinding was insufficiently described.
- ***High risk of bias:*** If no blinding or incomplete blinding of outcome assessors was performed.

Incomplete outcome data

- ***Low risk of bias:*** If missing data were unlikely to make treatment effects depart from plausible

values. This could be either (1) there were no drop-outs or withdrawals for all outcomes, or (2) the numbers and reasons for the withdrawals and drop-outs for all outcomes were clearly stated and could be described as being similar to both groups. Generally, the trial is judged as at a low risk of bias due to incomplete outcome data if drop-outs are less than 5%. However, the 5% cut-off is not definitive.

- ***Uncertain risk of bias:*** If there was insufficient information to assess whether missing data were likely to induce bias on the results.
- ***High risk of bias:*** If the results were likely to be biased due to missing data either because the pattern of drop-outs could be described as being different in the two intervention groups or the trial used improper methods in dealing with the missing data (e.g. last observation carried forward).

Selective outcome reporting

- ***Low risk of bias:*** If a protocol was published before or at the time the trial was begun and the outcomes specified in the protocol were reported on. If there is no protocol or the protocol was published after the trial has begun, reporting of serious adverse events will grant the trial a grade of low risk of bias.
- ***Uncertain risk of bias:*** If no protocol was published and the outcome of serious adverse events were not reported on.
- ***High risk of bias:*** If the outcomes in the protocol were not reported on.

Other risks of bias

- ***Low risk of bias:*** If the trial appears to be free of other components that could put it at risk of bias.
- ***Unclear risk of bias:*** If the trial may or may not be free of other components that could put it at risk of bias.
- ***High risk of bias:*** If there are other factors in the trial that could put it at risk of bias (including, Design-specific risk of bias, stopped early due to some data-dependent process including a formal-stopping rule, baseline imbalance, claimed fraudulent, blocked randomization in unblinded trials, differential diagnostic activity, contamination, inappropriate measurement instrument for outcomes, deviation from the study protocol unrelated to the clinical practice, authors conducted trials on the same topic, academic bias, for-profit bias, inappropriate

financial conflict of interest).

Overall risk of bias

- ***Low risk of bias:*** The trial will be classified as overall 'low risk of bias' only if all of the bias domains described in the above paragraphs are classified as 'low risk of bias'.
- ***High risk of bias:*** The trial will be classified as 'high risk of bias' if any of the bias risk domains described in the above are classified as 'unclear' or 'high risk of bias'.
- We will assess the domains 'blinding of outcome assessment', 'incomplete outcome data', and 'selective out- come reporting' for each outcome result. Thus, we can assess the bias risk for each outcome assessed in addition to each trial. Our primary conclusions will be based on the results of our primary outcome results with overall low risk of bias. Both our primary and secondary conclusions will be presented in the summary of findings tables.
