# Supplementary material for: Deep neuromuscular block for minimally invasive lung surgery: a protocol for a systematic review with meta-analysis and trial sequential analysis
Source: BMJ Open. 2022 May 23;12(5):e056816. doi: 10.1136/bmjopen-2021-056816 (PMC9131110; doi:10.1136/bmjopen-2021-056816)
Supplement: Author's manuscript [file bmjopen-2021-056816.draft_revisions.pdf]

BMJ Open is committed to open peer review. As part of this commitment we make the peer review history of every article we publish publicly available.

When an article is published we post the peer reviewers' comments and the authors' responses online. We also post the versions of the paper that were used during peer review. These are the versions that the peer review comments apply to.

The versions of the paper that follow are the versions that were submitted during the peer review process. They are not the versions of record or the final published versions. They should not be cited or distributed as the published version of this manuscript.

BMJ Open is an open access journal and the full, final, typeset and author-corrected version of record of the manuscript is available on our site with no access controls, subscription charges or pay-per-view fees (http://bmjopen.bmj.com).

If you have any questions on BMJ Open's open peer review process please email info.bmjopen@bmj.com

BMJ Open

Deep neuromuscular block for minimally invasive lung surgery: a protocol for a systematic review with meta-analysis and trial sequential analysis

Journal:	*BMJ Open*
Manuscript ID	bmjopen-2021-056816
Article Type:	Protocol
Date Submitted by the Author:	26-Aug-2021

Complete List of Authors:	Zheng, Jianqiao; Sichuan University West China Hospital, Department of Anesthesiology<br>Du, Li; University of Electronic Science and Technology of China, Department of Anesthesiology<br>Deng, Xiaoqian; Sichuan University West China Hospital, Department of Anesthesiology<br>Zhang, Lu; Sichuan University West China Hospital, Department of Anesthesiology<br>Wang, Jia; Sichuan University West China Hospital, Department of Anesthesiology<br>Chen, Guo; Sichuan University West China Hospital, Department of Anesthesiology

Keywords:	Cardiothoracic surgery < SURGERY, Adult anaesthesia < ANAESTHETICS, Adult surgery < SURGERY

SCHOLARONE™ Manuscripts

TITLE PAGE

Deep neuromuscular block for minimally invasive lung surgery: a protocol for a systematic review with meta-analysis and trial sequential analysis

Authors

Jianqiao Zheng[1] E-mail: zhjq1983@163.com

Li Du[2] E-mail: huaying-duli@163.com

Xiaoqian Deng[1] E-mail: 50058837@qq.com

Lu Zhang[1] E-mail: 304022514@qq.com

Jia Wang[1] E-mail: 19983157894@qq.com

*Guo Chen[1] E-mail: Anesthesiology_SCU@163.com

[1]Department of Anesthesiology, Sichuan University West China Hospital, No. 37th, Guoxue Alley, Wuhou District, Chengdu 610041, Sichuan, China.

[2]Department of Anesthesiology, Sichuan Cancer Hospital & Institute, Sichuan Cancer Center, School of Medicine, University of Electronic Science and Technology of China, No.55th, People's South Road, Chengdu 610041, Sichuan, China.

*Corresponding Author

Name: Guo Chen

E-mail: Anesthesiology_SCU@163.com

Phone: +86-28-8542-3593

Address: Department of Anesthesiology, Sichuan University West China Hospital, No. 37th, Guoxue Alley, Wuhou District, Chengdu 610041, Sichuan, China.

Words: 3284

For peer review only

ABSTRACT

Introduction Lung cancer is the leading cause of cancer death and surgical resection remains the primary therapy treatment. Minimally invasive surgical techniques of video-assisted and robotic-assisted thoracic surgery gradually become the popular surgical procedure. Adequate muscle relaxation by deep neuromuscular block (DNMB) is particularly necessary for minimally invasive surgery as to provide satisfactory surgical filed. In contrast, DNMB seem unnecessary for minimally invasive lung surgery (MILS) as one-lung ventilation usually provides acceptable surgical field. Therefore, the efficiency of DNMB for MILS remains controversial. Then, we will perform a protocol for a systematic review and meta-analysis to identify the clinical effect of DNMB for MILS.

Methods and analysis We will search PubMed, Web of Science, Cochrane Library, Ovid medline, Embase, China National Knowledge Infrastructure, Chinese BioMedical Literature, Wanfang and VIP databases from inception to September 2021, to identify randomised controlled trials using related keywords. Studies published in English or Chinese will be considered. Data synthesis will be performed using the RevMan 5.4 and Stata/MP16.0. We will present the outcome measures as relative risk (RR) with 95% confidence intervals (CIs) for dichotomous data and mean difference (MD) with 95% CIs for continuous data. The primary outcome will be the surgical conditions according to surgeon's

For peer review only

For peer review only - http://bmjopen.bmj.com/site/about/guidelines.xhtml

perspective. Secondary outcomes will be incidence of perioperative events and patients' postoperative recovery. Heterogeneity will be assessed by the χ^2 test and I^2 statistic. Data will be synthesised by either fixed-effects or random-effects models according to the I^2 value. The modified Jadad scale and trial sequential analysis will be used to assess the evidence quality and control the risks of random errors. Funnel plots and Egger's regression test will be used to assess the publication bias.

Ethics and dissemination Ethical approval was not required for this systematic review protocol. The findings will be disseminated through peer-reviewed publications.

Keywords deep neuromuscular block, minimally invasive, thoracoscopic, pulmonary, meta-analysis, randomized controlled trial.

PROSPERO registration number CRD42021254016

Strengths and limitations of the study

►This systematic review protocol follows the Preferred Reporting Items for Systematic Review and Meta-Analysis Protocols (PRISMA-P) guidelines to conduct a rigorous risk of bias assessment.

► Trial sequential analysis will be used to control the risks of false-positive by calculating the required information size for the outcomes.

► Funnel plots and Egger's regression test will be used to assess the publication bias.

►Subgroup analysis will be performed to assess the heterogeneity

For peer review only - http://bmjopen.bmj.com/site/about/guidelines.xhtml

according to patients' age, body mass index, and type of MILS.

INTRODUCTION

Lung cancer remains the leading cause of cancer death, with an estimated 1.8 million new deaths in 2020, accounting 18% of the total cancer deaths according to Global Cancer Statistics 2020. [1] Lung cancer is the second most commonly cancer with an estimated 2.2 million new cases in 2020, representing 11.4% of all cancer cases. [1] As the high incidence and mortality, the treatment of the lung cancer is a global challenge.

Surgical resection remains the primary therapy in the treatment of lung cancer. Since the 1990s, minimally invasive surgical (MIS) techniques of video-assisted thoracic surgery (VATS) and robotic-assisted thoracic surgery (RATS) have been applied in the diagnosis and treatment of intrathoracic diseases. [2-4] Growing experience with minimally invasive lung surgery (MILS), combined with the improvement in video technology and instrumentation, have allowed the conventional thoracotomy gradually replaced by MILS over recent years. [5-8]

Recent literature suggests that MILS was equivalent to open thoracotomy on long-term survival and overall oncologic efficacy, even with a better short-term survival.[9-15] MIS approach is still the favored surgical procedure in that it offers many advantages, including less trauma and pain, faster recovery, fewer complications, lower immunological responses, and a shorter hospitalization period. [16-21] In addition, it is

For peer review only - http://bmjopen.bmj.com/site/about/guidelines.xhtml

associated with a higher tolerance to postoperative adjuvant therapy, mitigates or ameliorates the postoperative decline of health-related functional status. [22-25]

Adequate muscle relaxation by deep neuromuscular block (DNMB) is mandatory for most surgical procedures, and particularly for MIS techniques. [26-28] MILS involves areas adjacent to major blood vessels and can trigger intraoperative body movement, cough, and diaphragm movement.[29] Moreover, the diaphragm is the most resistant muscle to neuromuscular blocking agents (NMBAs), movement of the diaphragm can interfere with the surgical procedure. DNMB can inhibit response to carinal stimulation and prevent bucking and coughing during surgical procedures. [30-32] In addition, it can reduce the peak pressure and plateau pressure, improve lung compliance and peripheral oxygen saturation during one-lung ventilation. [33]

There is still a controversy on the clinical benefit of maintaining DNMB for MILS, because DNMB seems unnecessary as the ribcage provides thoracic support and one-lung ventilation usually provides a satisfactory surgical field. In addition, the risk of residual neuromuscular block (RNMB) is estimated to occur in 26% to 88% of patients undergoing general anesthesia, and this incidence is inevitably increased after DNMB. [34 35] Numerous clinical studies have documented that postoperative RNMB has the potential risk to increase the incidence of postoperative pulmonary

For peer review only - http://bmjopen.bmj.com/site/about/guidelines.xhtml

complications (such as airway obstruction, aspiration, and hypoxia), the odds of hospital readmission intensive care unit admission and the hospital length of stay. [36-40]

Hence, the clinical benefits of DNMB for MILS remain controversial. Therefore, it is necessary to conduct a systematic review and meta-analysis to analyze the clinical efficacy of DNMB on MILS. Outcomes of this systematic review will provide evidence for better clinical decision making and possible directions for further clinical trials.

Objectives

We are conducting this protocol of systematic review and meta-analysis to determine the clinical efficacy of DNMB on surgical conditions of MILS according to the surgeon's perspective. Patients' postoperative recovery and the incidence of perioperative events will also be identified. Furthermore, we will use trial sequential analysis (TSA) to confirm the reliability of the results.

METHODS AND ANALYSIS

Study design

Our review protocol has been registered with PROSPERO (registration number: CRD 42021254016). This protocol was prepared according to the guidelines of Preferred Reporting Items for Systematic Reviews and Meta-Analyses Protocols (PRISMA-P) guidelines.[41] The systematic review and meta-analysis will be conducted in accordance with

For peer review only - http://bmjopen.bmj.com/site/about/guidelines.xhtml

the Cochrane Handbook and reported according to the PRISMA statement. [42 43] The study is expected to begin searching in September 2021 and end in December 2021.

Inclusion/exclusion criteria for study selection

Types of studies

We will include all randomized controlled trials (RCTs) that evaluated the DNMB for MILS. Only studies published in English or Chinese will be included.

Studies with the following situations will be excluded: (1) studies without control group; (2) studies compared DNMB by different kinds of NMBAs or compared DNMB surgery with NMBAs-free surgery; (3) studies with incomplete, incorrect data or the research data could not be used for statistical analysis; (4) studies were abstracts from conferences, editorials, duplicate publications, letters, reviews, observational studies, and retrospective studies.

Types of participants

Adult Participants (age ≥18 years old) undergoing any kind of MILS (including thoracoscopic surgery, VATS or RATS) with DNMB will be included. There will be no limits on study participants in terms of gender, ethnicity, and body mass index (BMI).

Types of interventions/controls

In the intervention group, participants had to receive DNMB [defined

For peer review only - http://bmjopen.bmj.com/site/about/guidelines.xhtml

as a train-of-four (TOF) count of zero or a post-tetanic count (PTC) count of 1-5] throughout the MILS.[44 45]

The control group will be the participants received shallow NMB (defined as a TOF ratio of 0.5 or a TOF count > 2), moderate NMB (defined as PTC>5 or TOF count=1-2), on-demand NMB (defined as neuromuscular antagonists was given on demand) or standard NMB (defined as neuromuscular antagonists was given as indication) throughout the MILS.[46-49]

Types of outcome measures

Primary outcomes

The primary outcome will be the surgical conditions of the MILS according to the surgeon's perspective. Surgical conditions were evaluated as surgical rating scale or the percentage of patients with clinically acceptable surgical conditions (Clinically acceptable surgical conditions will be defined as Acceptable, Good or Optimal conditions) (table 1).[50]

Table 1 Surgical rating scale (SRS)

SRS category (scale)	Conditions Description
Extremely poor conditions (Score 1)	The surgeon is unable to work because of coughing or of the inability to obtain a visible field because of inadequate muscle relaxation.
Poor conditions (Score 2)	There is a visible field, but the surgeon is severely hampered by inadequate muscle relaxation with continuous muscle contractions, movements, or both.
Acceptable conditions (Score 3)	There is a wide visible field but muscle contractions, movements, or both occur regularly
Good conditions (Score 4)	A wide working field with sporadic muscle contractions, movements, or both
Optimal conditions (Score 5)	A wide visible working field without any movement or contractions.

For peer review only - http://bmjopen.bmj.com/site/about/guidelines.xhtml

Secondary outcomes

1. Incidence of perioperative events including:

►Incidence of intraoperative events: defined as body movement, coughing, and breathing against the ventilator (with the aid of airway pressure monitoring and capnography).

►Incidence of postoperative pulmonary complications: defined as the composite of any of respiratory infection, respiratory failure, pleural effusion, atelectasis, or pneumothorax.

2. Patients' postoperative recovery

►Recovery time of neuromuscular block: defined as the time from administration of the reversal agent to the achievement of a TOF ratio of 0.9.

►The time from the end of surgery to discharge from the operating room to the recovery room.

Exploratory outcomes

1. Perioperative arterial blood gas including: Partial arterial oxygen pressure (PaO_2) or Oxygenation index (defined as PaO_2/fraction of inspired oxygen), Partial arterial carbon dioxide pressure ($PaCO_2$).
2. Postoperative pain visual analog scale (VAS) grade.
3. Postoperative cardiac complications including arrhythmia, cardiac tamponade, heart failure and so on.
4. Hospitalization time

For peer review only - http://bmjopen.bmj.com/site/about/guidelines.xhtml

Search strategy

We will search English and Chinese electronic databases from inception to September 2021 for published literatures. English database including PubMed, Web of Science, Cochrane Library, Ovid MEDLINE and Embase. Chinese database including China National Knowledge Infrastructure (CNKI), Chinese BioMedical Literature (CBM), Wanfang database and VIP Database. We will also scrutinize the reference lists of each literature and trial registry database (WHO International Clinical Trials Registry Platform and Clinical Trials.gov) for missing studies and unpublished or ongoing clinical trials. After data extraction, we will ask the corresponding authors of the included literatures for more grey literature to avoid potential missing as much as possible.

The search strategy for PubMed (as an example) is shown in table 2. The following search terms will be used: deep neuromuscular block, minimally invasive, thoracoscopic, video assisted, robotic assisted, pulmonary, and randomized controlled trial. The search terms will be translated into Chinese for study identification in Chinese databases. We will perform a new search in the databases to check if any studies were published during the elaboration of the systematic review before the final publication. The preliminary search strategy is given in (online supplementary additional file 1).

For peer review only - http://bmjopen.bmj.com/site/about/guidelines.xhtml

Table 2 Search strategy for PubMed

No	Search terms
#1	“Neuromuscular blockade"[MeSH] OR neuromusc*[tiab] OR “muscle relaxation” [MeSH]
#2	Deep[tiab] OR profound[tiab] OR intense[tiab] OR extreme[tiab] OR depth[tiab]
#3	“Pulmonary” [Mesh] OR “Lung” [Mesh] OR Pulmonary [tiab] OR Lung [tiab]
#4	“Surgical Procedures Operative” [Mesh] OR “Microsurgery” [Mesh] OR “Surgical Procedures Minimally Invasive” [Mesh] OR Minimally Invasive Surgery[tiab] OR MIS [tiab] OR Minimal Access Surgical Procedures [tiab]OR Minimal Surgical Procedures[tiab] OR Minimally Invasive Surgical Procedures [tiab] OR Minimal Surgical Procedure[tiab] OR minimally invasive surgical procedure [tiab] OR minimal access surgical procedure[tiab]
#5	“Thoracic surgery, Video-Assisted” [Mesh] or Surgeries, Video-Assisted Thoracic [af] or Surgery, Video-Assisted Thoracic [af] or Thoracic Surgeries, Video-Assisted [af] or Thoracic surgery, Video-Assisted [af] or Video-Assisted Thoracic Surgeries [af] or Video-Assisted Thoracic Surgery [af] or Surgeries, Video-Assisted Thoracoscopic [af] or Surgery, Video-Assisted Thoracoscopic [af] or Thoracoscopic Surgeries, Video-Assisted [af] or Thoracoscopic Surgery, Video-Assisted [af] or Video Assisted Thoracoscopic Surgery [af] or Video Assisted Thoracoscopic Surgeries [af] or Video-Assisted Thoracic Surgery [af] or Video Assisted Thoracic Surgery [af] or Surgery, Thoracic, Video-Assisted [af] or VATS [af] or VATSs [af].
#6	“Robotics” [MeSH] OR robot* [tiab] OR computer guid*[tiab] OR computer-guid*[tiab] OR computer-assisted[tiab] OR computer assisted [tiab]OR da Vinci [tiab]OR Zeus [tiab]OR telesurgery[tiab]
#7	#1 AND #2 AND #3
#8	#4 OR #5 OR #6
#9	“Controlled clinical trial” [Publication Type] OR “randomized controlled trial” [Publication Type] OR “randomized” [Title/Abstract] OR “randomized” [Title/Abstract] OR “Placebo” [Title/Abstract] OR “randomly” [Title/Abstract] OR “Clinical trial” [Title]
#10	“animals” [MeSH] NOT (“human” [MeSH] AND “animals” [MeSH])
#11	#7and #8 and #9 not #10

For peer review only - http://bmjopen.bmj.com/site/about/guidelines.xhtml

Data collection and analysis

Selection of studies

Two reviewers (Z-JQ and WJ) will be required to screen the retrieved studies independently. Briefly, they will exclude duplicate studies and those not matching the inclusion criteria by reading title and abstracts. After reading the full text of each study, studies meeting the inclusion criteria will be selected. Any disagreements will be resolved through discussion with a third reviewer (DL). A fourth reviewer (CC) will check all procedures before approving the data extraction. Details of the entire study selection procedure will be shown in the PRISMA flow diagram (figure 1).

Data extraction

Two reviewers (Z-JQ and ZL) will extract data from the included studies independently following a data acquisition Microsoft Excel software. Required information including demographic data, type of MILS, inclusion/exclusion criteria, Level of NMB during MILS (definition and measurement), outcome indicators (primary, secondary, and exploratory outcomes), etc. Information about study design (such as randomization, allocation concealment, blinding methods, data collection and statistical analysis, outcome reporting) will also be recorded for the next step quality assessment. Result data will be recorded as mean± SD for continuous variable, and proportion of participants with percentage for dichotomous

For peer review only - http://bmjopen.bmj.com/site/about/guidelines.xhtml

data. If necessary, a third reviewer (D-XQ) will double-check the data to ensure consistency. If information and data are missing or incomplete in any study, we will contact with the corresponding authors of the literatures to obtain the original data by email. If necessary, we will extract numerical data from graphs using Adobe Photoshop as described by Gheibi et al.[51] Detailed list of information and data to be extracted is presented as table 3.

Table 3 Data and information extraction schedule

Subject	Content
Publication information	Name of the first author; contact email; publish year; country; corporate sponsorship.
Participant	Sample size; Age; Sex; Height and weight or body mass index (BMI); American Society of Anesthesiologists (ASA) physical status classification levels; Type of MILS; Inclusion and exclusion criteria if necessary.
Intervention	Level of NMB (moderate NMB; shallow NMB; on-demand NMB or standard NMB); Assessment of the NMB level (equipment of neuromuscular function monitor; monitor position); Type of neuromuscular blocking agents (NMBAs); Dose and administration of NMBAs; Administration of NMBAs antagonist (Sugammadex or Neostigmine).
Control	Definition of the DNMB; Assessment of the DNMB (equipment for neuromuscular function monitor; monitor position); Type of neuromuscular blocking agents (NMBAs); Dose and administration of NMBAs; Administration of NMBAs antagonist (Sugammadex or Neostigmine).
Outcome	Primary outcome (Surgical rating scale or the percentage of patients with clinically acceptable surgical conditions); Secondary outcome measurements (Perioperative events; Patients' postoperative recovery indicators); Exploratory outcomes (Perioperative arterial blood gas; Postoperative VAS grade; Postoperative cardiac complications; Hospitalization time).
Study design	Application of randomization and blinding; Description about allocation concealment; Statistical analysis; Sample size calculation; Outcome reporting.
Other information	Intraoperative temperatures; Bispectral index (BIS) values; Time or condition of tracheal intubation and extubation; Type of anesthesia maintenance technique (Inhalation anesthesia; Total intravenous anesthesia; or both); Duration of surgery and anesthesia.

For peer review only - http://bmjopen.bmj.com/site/about/guidelines.xhtml

Quality assessment

Two reviewers (WJ and ZL) will assess the risk of bias in the included studies with the guidance of Cochrane Handbook independently.[52] The Cochrane Collaboration's tool covers six aspects: randomization; allocation concealment; blinding (including blinding of participants and personnel; blinding of outcome assessment); data collection and statistical analysis; selective reporting and other bias. The risk will be divided into three levels (low risk, unclear and high risk) in accordance with the item in the checklist. If any disagreements, the risk assignment will be settled through discourse. If this discourse is not conducive, discrepancies will be resolved by a third reviewer (CC).

Evidence grade evaluation

We will apply the modified Jadad scale to evaluate the quality of each outcome's evidence grade.[53 54] Evidence grade evaluation of the included studies will be conducted in eight items: randomization (with score 0-2), blinding (with score 0-2), withdrawals and dropouts (with score 0-1), inclusion and exclusion criteria (with score 0-1), adverse effects (with score 0-1), and statistical analysis (with score 0-1) (table 4). Scale scores for each study could range from 0 to 8 points, with higher scores indicating better quality: score 1-3 signified low-quality; score 4-8 signified high-quality.

For peer review only - http://bmjopen.bmj.com/site/about/guidelines.xhtml

Table 4 The modified Jaded Scale

Items	Score
1. Was the study described as randomized?	
Yes	1
No	0
2. Was the method of randomization appropriate?	
Yes	1
No	-1
Not described	0
3. Was the study described as blinded?	
Yes (Double-blind)	1
Yes (Single-blind)	0.5
No	0
4. Was the method of blinding appropriate?	
Yes	1
No	-1
Not described	0
5. Was there a description of withdrawals and dropouts?	
Yes	1
No	0
6. Was there a clear description of the inclusion and exclusion criteria?	
Yes	1
No	0
7. Was the method used to assess adverse effects described?	
Yes	1
No	0
8. Was the method of statistical analysis described?	
Yes	1
No	0

Measures of treatment effect

For continuous outcome data, the mean differences (MDs) or the standardised mean difference (SMDs) with 95% confidence intervals (CIs)

For peer review only - http://bmjopen.bmj.com/site/about/guidelines.xhtml

will be used for analysis. For dichotomous data, the relative risks (RR) with 95% CIs will be used for analysis.

Assessment of heterogeneity

We will calculate I^2 to test heterogeneity for each pooled result with Review Manager version 5.4 (Rev Man, Cochrane Collaboration, Oxford, UK). Statistical heterogeneity will be assessed by standard χ^2 test (α=0.1) and I^2 test. If the $p \geq 0.1$, and if $I^2 \leq 50\%$, fixed-effects model will be used. If the $p<0.1$ or the $I^2>50\%$, random-effects models will be applied. When the heterogeneity is statistically significant, we will conduct a subgroup analysis to investigate the possible sources of heterogeneity according to the patient's characteristics (such as age and BMI) and type of MILS. If the $I^2>75\%$, a meta-analysis will not be performed and a narrative, qualitative summary will be provided.

Trial Sequential Analysis

Trial Sequential Analysis (TSA) will be performed by Stata/MP 16.0 (Stata Corp, College Station, TX, USA) to control the risks of false-positive by calculating the required information size (RIS).[55-57] RIS is the number of participants needed in a meta-analysis to detect or reject a certain intervention effect. [58 59] RIS and information size for each outcome will be calculated. In addition, the cumulative Z-curve's breach of relevant trial sequential to monitor boundaries will be calculated for all outcomes.[58 59]

For peer review only

For continuous outcomes, we will use the observed SD, a mean difference of the observed SD/2, an alpha of 2.5% and a beta of 10% for primary and secondary outcomes in the TSA.[60] For dichotomous outcomes, we will use the proportion of participants with an outcome in the control group, a relative risk reduction of 0.20, and an alpha of 0.025 and a beta of 0.10 in the TSA. [61] TSA will be performed using the TSA program version 0.9.5.10 Beta (http://www.ctu.dk/tsa).[62]

Subgroup analysis

We will further explain the results with analysis of subgroups or subsets. If sufficient trials are available, data from different age, different body mass index (BMI) and different type of MILS will be analysed separately.

► Different Age (DNMB for MILS in patients with different age as follows: 18 years≤ Patients < 60 years; 60 years≤ Patients < 75 years; Patients≥75 years).

► Different type of MILS (DNMB for Video assistant thoracoscopic lung surgery; DNMB for Robotic-assistant thoracoscopic lung surgery).

► Different BMI (DNMB for MILS in patients with different BMI as follows: BMI<25.0 kg/m^2; 25.0 kg/m^2≤BMI < 30 kg/m^2; BMI≥30kg/m^2).

Sensitivity analysis

After analysis of subgroups or subsets, sensitivity analysis will be used to evaluate how uncertain assumptions of data and usage affect the

For peer review only - http://bmjopen.bmj.com/site/about/guidelines.xhtml

robustness of the combined results. We will exclude low quality studies, re-analyse the included studies, and assess whether there are significant differences between the combined effects. If necessary, we will remove the included studies one by one to observe whether the pooled estimations are stable or not. Significant changes may indicate significant heterogeneity among studies.

Assessment of publication biases

The potential publication bias will be statistically analysed using funnel plots analysis and Egger's regression test while no less than 10 original studies are involved for an outcome. [63 64] The trim-and-fill analysis will also be done to adjust any potential publication bias, as it is basing on the assumption that the effect sizes of all the studies are normally distributed around the center of a funnel plot in the absence of publication bias. [65] Publication biases will be performed by Stata/MP 16.0 (Stata Corp, College Station, TX, USA).

Patient and public involvement statement

Patients or the public were not involved in the design, or conduct, or reporting, or dissemination plans of our research.

DISCUSSION

This systematic review will provide an overview of the current state of evidence concerning the clinical efficacy of DNMB for MILS. We will examine the effect of DNMB on surgical conditions according to the

For peer review only - http://bmjopen.bmj.com/site/about/guidelines.xhtml

surgeon's perspective. In addition, we will investigate the efficacy of DNMB on patients' postoperative recovery and postoperative complications. To the best of our knowledge, this will be the first systematic review concerning this topic. Outcomes of this systematic review will provide evidence for better clinical decision making on the management of the NMB and patient care during MILS.

This systematic review protocol follows the Preferred Reporting Items for Systematic Review and Meta-Analyses Protocols (PRISMA-P) guidelines. Strengths of our systematic review include: First, comprehensive search in English and Chinese databases. Second, multivariable analysis (including assessment of study quality, subgroup analysis, sensitivity analysis, trial sequential analysis and Egger's regression test) will be used to minimize the confounding bias. Third, screening, data extraction and quality assessment will be performed by two independent reviewers according to guidelines.

There are also limitations to our analysis. First, studies with different NMBAs and NMBAs antagonist (Sugammadex or Neostigmine) will be included, resulting in potential heterogeneity. Second, number of studies with eligible data for subgroup analyses may be limited. Third, the sample size in each study may be small. Fourth, another limitation may be the current lack of high-level evidence, such as well-designed randomized controlled trials with double-blind. Thus, we will use rigorous methods

For peer review only

For peer review only - http://bmjopen.bmj.com/site/about/guidelines.xhtml

such as the TSA and trim-and-fill analysis in the data analysis and will meta-analysis the outcomes as appropriate.

ETHICS AND DISSEMINATION

Ethical approval was not required for this systematic review protocol. The findings will be disseminated through peer-reviewed publications.

Timelines

Formal screening of search results will begin in September 2021. Data extraction will begin in October 2021. The project is due to complete in December 2021.

Author Contributors

Z-JQ and DL conceived the idea for this systematic review. All authors (Z-JQ, DL, WJ, ZL, D-XQ, CC) developed the methodology for the systematic review. The manuscript was drafted by Z-JQ, DL, and revised by all authors. D-XQ and CC will screen potential studies, perform duplicate independent data abstraction. Z-JQ and ZL will undertake risk of bias assessment and assess the evidence quality. Z-JQ and DL will conduct the data synthesis. All authors contributed to the research and agreed to be responsible for all aspects of the work.

Funding

None

Competing interests

None declared.

For peer review only - http://bmjopen.bmj.com/site/about/guidelines.xhtml

Data availability statement

Not applicable for this protocol.

Patient consent for publication

No patient involved.

Provenance and peer review

Not commissioned; externally peer reviewed.

REFERENCES

1 Sung H, Ferlay J, Siegel RL, *et al*. Global Cancer Statistics 2020: GLOBOCAN Estimates of Incidence and Mortality Worldwide for 36 Cancers in 185 Countries. *CA Cancer J Clin* 2021; 71:209-49.

2 McKenna RJ Jr, Houck W, Fuller CB. Video-assisted thoracic surgery lobectomy: experience with 1,100 cases. *Ann Thorac Surg* 2006;81: 421-5; discussion 425-6.

3 Fahim C, Hanna W, Waddell T, *et al*. Robotic-assisted thoracoscopic surgery for lung resection: the first Canadian series. *Can J Surg*2017; 60:260-65.

4 Ujiie H, Gregor A, Yasufuku K. Minimally invasive surgical approaches for lung cancer. *Expert Rev Respir Med* 2019;13(6):571-78.

5 Sihoe AD. Uniportal video-assisted thoracic (VATS) lobectomy. Ann *Cardiothorac Surg* 2016; 5:133-44.

6 Hernandez-Arenas LA, Lin L, Yang Y, *et al*. Initial experience in uniportal subxiphoid video-assisted thoracoscopic surgery for major lung resections. *Eur J Cardiothorac Surg* 2016;50: 1060-66.

7 Ujiie H, Yasufuku K. New era of "resection of the carina and lower trachea. *J Thorac Dis* 2017; 9:4932-36.

8 Ujiie H, Kato T, Hu HP, *et al*. A novel minimally invasive near-infrared thoracoscopic localization technique of small pulmonary nodules: A phase

For peer review only - http://bmjopen.bmj.com/site/about/guidelines.xhtml

I feasibility trial. *J Thorac Cardiovasc Surg* 2017; 154:702-11.

9 Boffa DJ, Kosinski AS, Furnary AP, *et al*. Minimally Invasive Lung Cancer Surgery Performed by Thoracic Surgeons as Effective as Thoracotomy. *J Clin Oncol* 2018; 36:2378-85.

10 Long H, Tan Q, Luo Q, *et al*. Thoracoscopic surgery versus thoracotomy for lung cancer: short-term outcomes of a randomized trial. *Ann Thorac Surg* 2018; 105:386-92.

11 Weksler B, Tavares J, Newhook TE, *et al*. Robot-assisted thymectomy is superior to transsternal thymectomy. *Surg Endosc* 2012; 26:261-6.

12 Han KN, Kim HK, Choi YH. Midterm outcomes of single port thoracoscopic surgery for major pulmonary resection. *PloS one* 2017;12: e0186857.

13 Shen H, Wang X, Nie Y, *et al*. Minimally invasive surgery versus thoracotomy for resectable stage II and III non-small-cell lung cancers: a systematic review and meta-analysis. *Eur J Cardiothorac Surg* 2020; 59: 940-50.

14 O'Sullivan KE, Kreaden US, Hebert AE, *et al*. A systematic review and meta-analysis of robotic versus open and video-assisted thoracoscopic surgery approaches for lobectomy. *Interact Cardiovasc Thorac Surg* 2019; 28:526-34.

15 Yang CJ, Kumar A, Klapper JA, *et al*. A national analysis of long-term survival following thoracoscopic versus open lobectomy for stage I non-small-cell lung cancer. *Annals of surgery* 2019; 269:163-171.

16 O'Sullivan KE, Kreaden US, Hebert AE, *et al*. A systematic review and meta-analysis of robotic versus open and video-assisted thoracoscopic surgery approaches for lobectomy. *Interact Cardiovasc Thorac Surg* 2019; 28:526-34.

17 Li Z, Xia M, Liu C, *et al*. A meta-analysis of minimally invasive surgery versus thoracotomy for centrally located non-small cell lung cancer. *J Thorac Dis* 2021; 13:252-61.

18 Bendixen M, Jørgensen OD, Kronborg C, *et al*. Postoperative pain and

For peer review only

quality of life after lobectomy via video-assisted thoracoscopic surgery or anterolateral thoracotomy for early stage lung cancer: a randomised controlled trial. *Lancet Oncol* 2016; 17: 836-44.

19 Swanson SJ, Miller DL, McKenna RJ Jr., et al. Comparing robot-assisted thoracic surgical lobectomy with conventional video-assisted thoracic surgical lobectomy and wedge resection: results from a multihospital database (Premier). *J Thorac Cardiovasc Surg* 2014;147: 929-37.

20 Paul S, Jalbert J, Isaacs AJ, et al. Comparative effectiveness of robotic-assisted vs thoracoscopic lobectomy. *Chest* 2014;146: 1505-12.

21 Guerrera F, Olland A, Ruffini E, *et al.* VATS lobectomy vs. open lobectomy for early-stage lung cancer: an endless question-are we close to a definite answer? *J Thorac Dis* 2019; 11:5616-8.

22 Swanson SJ, Herndon JE, D'Amico TA, et al. Video- assisted thoracic surgery lobectomy: report of CALGB 39802-a prospective, multi-institution feasibility study. *J Clin Oncol* 2007; 25:4993-7.

23 Cao C, Manganas C, Ang SC, *et al*. Video-assisted thoracic surgery versus open thoracotomy for non-small cell lung cancer: a meta-analysis of propensity score-matched patients. *Interact Cardiovasc Thorac Surg* 2013; 16:244-9.

24 Guerrera F, Olland A, Ruffini E, *et al*. VATS lobectomy vs. open lobectomy for early-stage lung cancer: an endless question-are we close to a definite answer? *J Thorac Dis* 2019;11: 5616-8.

25 Handy JR Jr. Minimally invasive lung surgery and postoperative quality of life. *Thorac Surg Clin* 2012;22:487-95.

26 Iwata H. Minimally invasive pulmonary surgery for lung cancer, up to date. *Gen Thorac Cardiovasc Surg* 2013; 61:449–54.

27 Barrio J, Errando CL, Miguel GS, *et al*. Effect of depth of neuromuscular blockade on the abdominal space during pneumoperitoneum establishment in laparoscopic surgery. *J Clin Anesth* 2016; 34:197-203.

28 Kim HJ, Lee K, Park WK, *et al*. Deep neuromuscular block improves the

For peer review only - http://bmjopen.bmj.com/site/about/guidelines.xhtml

surgical conditions for laryngeal microsurgery. *Br J Anaesth* 2015; 115:867-72.

29 Lesser TG, Schubert H, Güllmar D, *et al*. One-lung flooding reduces the ipsilateral diaphragm motion during mechanical ventilation. *Eur J Med Res* 2016; 21:9.

30 Gibson PG, Simpson JL, Ryan NM, *et al*. Mechanisms of cough. *Curr Opin Allergy Clin Immunol* 2014; 14:55-61.

31 Fernando PU, Viby-Mogensen J, Bonsu AK, *et al*. Relationship between posttetanic count and response to carinal stimulation during vecuronium-induced neuromuscular blockade. *Acta Anaesthesiol Scand* 1987;31: 593–6.

32 Dhonneur G, Kirov K, Motamed C, *et al.* Post-tetanic count at adductor pollicis is a better indicator of early diaphragmatic recovery than train-of-four count at corrugator supercilii. *Br J Anaesth* 2007;99: 376–9.

33 Casanova J, Piñeiro P, De La Gala F, *et al*. Deep versus moderate neuromuscular block during one-lung ventilation in lung resection surgery. *Rev Bras Anestesiol* 2017;67: 288-93.

34 Fortier LP, McKeen D, Turner K, *et al.* The RECITE study: a Canadian Prospective, Multicenter Study of the incidence and severity of residual neuromuscular blockade. *Anesth Analg* 2015; 121:366-72.

35 Raval AD, Uyei J, Karabis A, *et al.* Incidence of residual neuromuscular blockade and use of neuromuscular blocking agents with or without antagonists: A systematic review and meta-analysis of randomized controlled trials. *J Clin Anesth* 2020; 64:109818.

36 Kheterpal S, Vaughn MT, Dubovoy TZ, *et al*. Sugammadex versus Neostigmine for Reversal of Neuromuscular Blockade and Postoperative Pulmonary Complications (STRONGER): A Multicenter Matched Cohort Analysis. *Anesthesiology* 2020; 132:1371-81.

37 Cammu G. Residual Neuromuscular Blockade and Postoperative Pulmonary Complications: What Does the Recent Evidence Demonstrate?

For peer review only - http://bmjopen.bmj.com/site/about/guidelines.xhtml

Curr Anesthesiol Rep 2020; 27:1-6.

38 Grabitz SD, Rajaratnam N, Chhagani K, *et al*. The Effects of Postoperative Residual Neuromuscular Blockade on Hospital Costs and Intensive Care Unit Admission: A Population-Based Cohort Study. *Anesth Analg* 2019; 128:1129-36.

39 Stawicki N, Gessner P. Residual Neuromuscular Blockade in the Critical Care Setting. *AACN Adv Crit Care*2018;29: 15-24.

40 Saager L, Maiese EM, Bash LD, *et al*. Incidence, risk factors, and consequences of residual neuromuscular block in the United States: The prospective, observational, multicenter RECITE-US study. *J Clin Anesth* 2019; 55:33-41.

41 Shamseer L, Moher D, Clarke M. Preferred reporting items for systematic review and meta-analysis protocols (PRISMA-P) 2015: elaboration and explanation. *BMJ* 2015; 350: g7647.

42 Higgins JPT TJ, Chandler J, Cumpston M. Cochrane Handbook for systematic reviews of interventions version 6.1 (updated September 2020). The Cochrane collaboration, 2020. Available: www.training. cochrane.org/ handbook.

43 Liberati A, Altman DG, Tetzlaff J. The PRISMA statement for reporting systematic reviews and meta-analyses of studies that evaluate healthcare interventions: explanation and elaboration. *BMJ* 2009;339: b2700.

44 Groudine SB, Soto R, Lien C, *et al*. A randomized, dose-finding, phase II study of the selective relaxant binding drug, Sugammadex, capable of safely reversing profound rocuronium-induced neuromuscular block. *Anesth Analg* 2007; 104:555-62.

45 Dhonneur G, Kirov K, Motamed C, *et al*. Post-tetanic count at adductor pollicis is a better indicator of early diaphragmatic recovery than train-of-four count at corrugator supercilii. *Br J Anaesth* 2007; 99:376-9.

46 Choi ES, Oh AY, Seo KS, *et al.* Optimum dose of neostigmine to reverse shallow neuromuscular blockade with rocuronium and cisatracurium.

For peer review only - http://bmjopen.bmj.com/site/about/guidelines.xhtml

Anaesthesia 2016; 71:443-9.

47 Fuchs-Buder T, Meistelman C, Alla F, *et al*. Antagonism of low degrees of atracurium-induced neuromuscular blockade: dose-effect relationship for neostigmine. *Anesthesiology* 2010; 112:34-40.

48 Özdemir-van Brunschot DMD, Braat AE, van der Jagt MFP, *et al*. Deep neuromuscular blockade improves surgical conditions during low-pressure pneumoperitoneum laparoscopic donor nephrectomy. *Surg Endosc* 2018; 32:245-51.

49 Staehr-Rye AK, Rasmussen LS, Rosenberg J, *et al*. Surgical space conditions during low-pressure laparoscopic cholecystectomy with deep versus moderate neuromuscular blockade: A randomized clinical study. *Anesth Analg* 2014; 119:1084-92.

50 Martini CH, Boon M, Bevers RF, *et al*. Evaluation of surgical conditions during laparoscopic surgery in patients with moderate vs. deep neuromuscular block. *Br J Anaesth* 2014;112: 498-505.

51 Gheibi S, Mahmoodzadeh A, Kashfi K, *et al*. Data Extraction from Graphs Using Adobe Photoshop: Applications for Meta-Analyses. *Int J Endocrinol Metab* 2019;17: e95216.

52 44 Higgins JPT, Altman DG, Gøtzsche PC, *et al*. The Cochrane collaboration's tool for assessing risk of bias in randomised trials. *BMJ* 2011; 343: d5928.

53 Jadad AR, Moore RA, Carroll D, *et al*. Assessing the quality of reports of randomized clinical trials: is blinding necessary? *Control Clin Trials* 1996; 17:1-12.

54 Oremus M, Wolfson C, Perrault A, *et al*. Interrater reliability of the modified Jadad quality scale for systematic reviews of Alzheimer's disease drug trials. *Dement Geriatr Cogn Disord* 2001;12: 232-6.

55 Wetterslev J, Thorlund K, Brok J, *et al*. Estimating required information size by quantifying diversity in random-effects model meta-analyses. *BMC Med Res Methodol* 2009; 9:86.

For peer review only - http://bmjopen.bmj.com/site/about/guidelines.xhtml

56 Wetterslev J, Jakobsen JC, Gluud C. Trial sequential analysis in systematic reviews with meta-analysis. *BMC Med Res Methodol* 2017; 17:39.

57 Wetterslev J, Thorlund K, Brok J, *et al*. Trial sequential analysis may establish when firm evidence is reached in cumulative meta-analysis. *J Clin Epidemiol* 2008; 61: 64-75.

58 Imberger G, Thorlund K, Gluud C, *et al*. False-Positive findings in Cochrane meta-analyses with and without application of trial sequential analysis: an empirical review. *BMJ Open* 2016;6: e011890.

59 Brok J, Thorlund K, Gluud C, *et al*. Trial sequential analysis reveals insufficient information size and potentially false positive results in many meta-analyses. *J Clin Epidemiol* 2008; 61:763-9.

60 Norman GR, Sloan JA, Wyrwich KW. Interpretation of changes in health-related quality of life: the remarkable universality of half a standard deviation. *Med Care* 2003; 41:582-92.

61 Castellini G, Nielsen EE, Gluud C. Comment on: "Cell therapy for heart disease: Trial sequential analyses of two cochrane reviews". *Clin Pharmacol Ther* 2017; 102:21-4.

62 Thorlund K, Engstrøm J, Wetterslev J, *et al.* User manual for trial sequential analysis (TSA) Copenhagen trial unit, centre for clinical intervention research, Denmark; 2011.Available: http://www.ctu.dk/tsa [Accessed 2021].

63 Egger M, Davey Smith G, Schneider M, *et al*. Bias in meta-analysis detected by a simple, graphical test. *BMJ* 1997;315: 629-34.

64 Sterne JA, Gavaghan D, Egger M. Publication and related bias in meta-analysis: power of statistical tests and prevalence in the literature. *J Clin Epidemiol* 2000; 53:1119-29.

65 Mavridis D, Salanti G. How to assess publication bias: funnel plot, trim-and-fill method and selection models. *Evid Based Ment Health* 2014; 17:30.

Figure Legends

Figure 1. The PRISMA flow diagram.

For peer review only - http://bmjopen.bmj.com/site/about/guidelines.xhtml

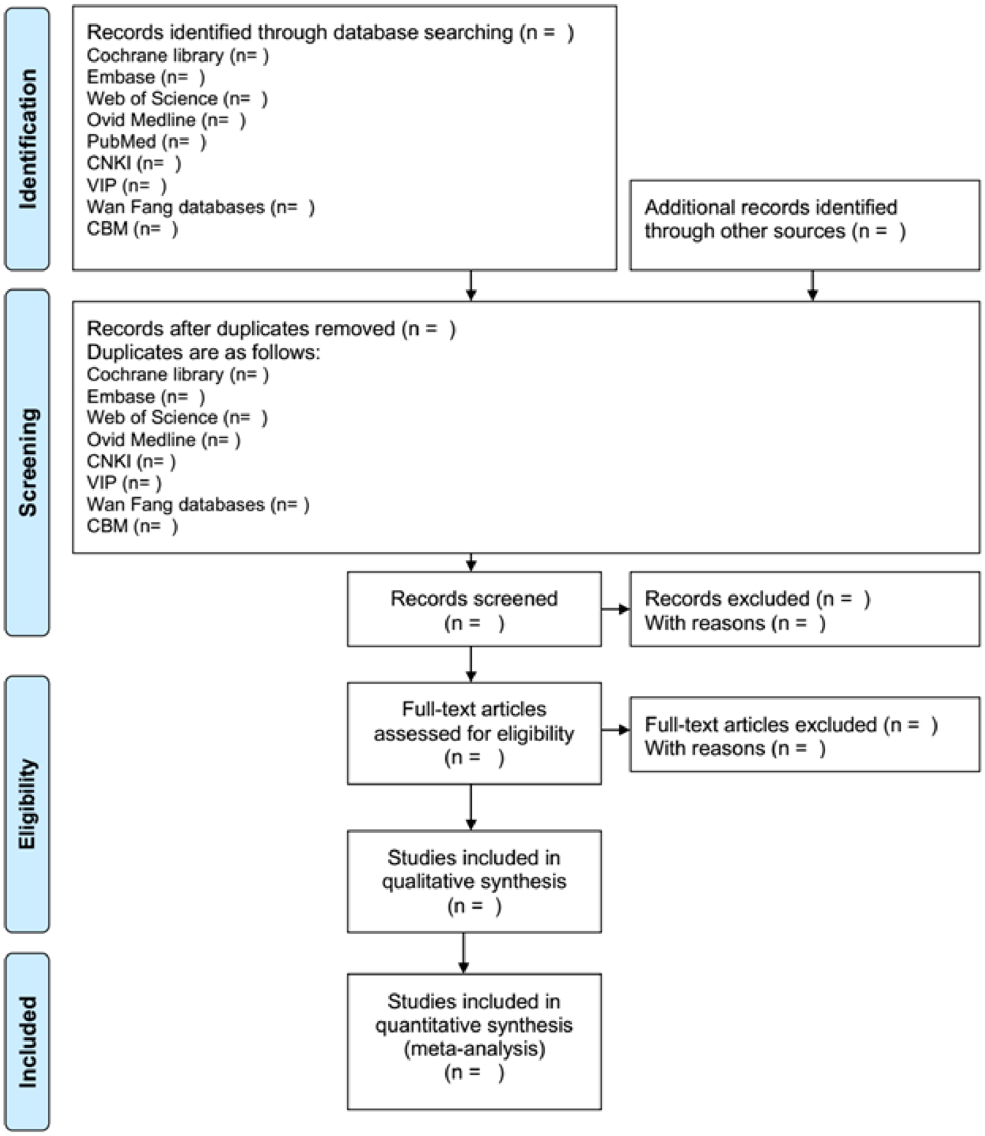
Identification
Records identified through database searching (n =)
Cochrane library (n=)
Embase (n=)
Web of Science (n=)
Ovid Medline (n=)
PubMed (n=)
CNKI (n=)
VIP (n=)
Wan Fang databases (n=)
CBM (n=)
Additional records identified through other sources (n =)
Screening
Records after duplicates removed (n =)
Duplicates are as follows:
Cochrane library (n=)
Embase (n=)
Web of Science (n=)
Ovid Medline (n=)
CNKI (n=)
VIP (n=)
Wan Fang databases (n=)
CBM (n=)
Records screened (n =)
Records excluded (n =)
With reasons (n =)
Eligibility
Full-text articles assessed for eligibility (n =)
Full-text articles excluded (n =)
With reasons (n =)
Studies included in qualitative synthesis (n =)
Included
Studies included in quantitative synthesis (meta-analysis) (n =)

Supplementary appendix 1: Search strategy

Search strategy of PubMed as follows:

#1 “neuromuscular blockade"[MeSH Terms] OR neuromusc*[tiab]” OR “muscle relaxation” [MeSH Terms]

#2 “Deep[tiab] OR profound[tiab] OR intense[tiab] OR extreme[tiab] OR depth[tiab]”

#3 “Pulmonary” [Mesh] OR “Lung” [Mesh] OR Pulmonary [tiab] OR Lung [tiab]

#4 “Surgical Procedures Operative” [Mesh] OR “Microsurgery” [Mesh] OR “Surgical Procedures Minimally Invasive” [Mesh] OR Minimally Invasive Surgery[tiab] OR MIS [tiab] OR Minimal Access Surgical Procedures [tiab]OR Minimal Surgical Procedures[tiab] OR Minimally Invasive Surgical Procedures [tiab] OR Minimal Surgical Procedure[tiab] OR minimally invasive surgical procedure [tiab] OR minimal access surgical procedure[tiab]

#5 “Thoracic surgery, Video-Assisted” [Mesh] or Surgeries, Video-Assisted Thoracic [af] or Surgery, Video-Assisted Thoracic [af] or Thoracic Surgeries, Video-Assisted [af] or Thoracic surgery, Video-Assisted [af] or Video-Assisted Thoracic Surgeries [af] or Video-Assisted Thoracic Surgery [af] or Surgeries, Video-Assisted Thoracoscopic [af] or Surgery, Video-Assisted Thoracoscopic [af] or Thoracoscopic Surgeries, Video-Assisted [af] or Thoracoscopic Surgery, Video-Assisted [af] or Video Assisted Thoracoscopic Surgery [af] or Video Assisted Thoracoscopic Surgeries [af] or Video-Assisted Thoracic Surgery [af] or Video Assisted Thoracic Surgery [af] or Surgery, Thoracic, Video-Assisted [af] or VATS [af] or VATSs [af].

#6 “Robotics” [MeSH] OR robot* [tiab] OR computer guid*[tiab] OR computer-guid*[tiab] OR computer-assisted[tiab] OR computer assisted [tiab]OR da Vinci [tiab]OR Zeus [tiab]OR telesurgery[tiab]

#7 #1 AND #2 AND #3

#8 #4 OR #5 OR #6

#9 “controlled clinical trial” [Publication Type] OR “randomized controlled trial”

For peer review only

[Publication Type] OR “randomized” [Title/Abstract] OR “randomized” [Title/Abstract] OR “Placebo” [Title/Abstract] OR “randomly” [Title/Abstract] OR “Clinical trial” [Title]

#10 (animals [MeSH Terms]) NOT ((human [MeSH Terms]) AND (animals [MeSH Terms]))

#11 #7and #8 and #9 not #10

Search strategy of Cochrane library as follows:

#1 MeSH descriptor: [neuromuscular blockade] explode all trees

#2 MeSH descriptor: [muscle relaxation] explode all trees

#3 (neuromusc*): ti,ab,kw

#4 #1 or # 2 or # 3

#5 (Deep): ti,ab,kw or (profound):ti,ab,kw or (intense):ti,ab,kw or (extreme):ti, ab,kw or (depth):ti,ab,kw

#6 #4 and #5

#7 MeSH descriptor: [Pulmonary] explode all trees

#8 MeSH descriptor: [Lung] explode all trees

#9 #7 or # 8

#10 #6 and #9

#11 MeSH descriptor: [Surgical Procedures Operative] explode all trees

#12 MeSH descriptor: [Thoracic surgery, Video-Assisted] explode all trees

#13 MeSH descriptor: [Microsurgery] explode all trees

#14 MeSH descriptor: [Surgical Procedures Minimally Invasive] explode all trees

#15 MeSH descriptor: [Robotics] explode all trees

#16 (surgery or surgical* or Video-Assisted* or Video Assisted* or Video* or Thorac* or VATS): ti,ab,kw

#17 (robot*): ti,ab,kw

#18 (computer guid* OR computer-guid* OR computer-assisted OR computer assisted): ti,ab,kw

For peer review only

#19 (da Vinci OR Zeus OR telesurgery): ti,ab,kw

#20 #11 or # 12 or #13 or # 14 or #15 or # 16 or #17 or # 18 or #19

#21 #10 and # 20

#22 (controlled clinical trial):pt or (randomized controlled trial):pt or (random*): ti,ab,kw or (Clinical trial):ti,ab,kw

#23 #21 and #22

Search strategy of Web of Science as follows:

#1 TS= (neuromuscular blockade or neuromusc* or muscle relaxation)

#2 TS= (Deep or profound or intense or extreme or depth)

#3 TS= (Pulmonary or Lung)

#4 TS= (Surgical Procedures Operative or Thoracic surgery, Video-Assisted or Microsurgery or Surgical Procedures Minimally Invasive or Robotics)

#5TS= (surgery or surgical* or Video-Assisted* or Video Assisted* or Video* or Thorac* or VATS or robot* or computer guid* or computer-guid* or computer-assisted or computer assisted or da Vinci or Zeus or telesurgery)

#6 #4 OR #5

#7 #1 and #2 and #3 and #6

#8 TS= (random* or Clinical trial)

#9 #7 and #8

Search strategy for Ovid Medline as follows:

#1 exp neuromuscular blockade/ or exp muscle relaxation/ or neuromusc*.mp.

#2 (Deep or profound or intense or extreme or depth) .mp.

#3 exp pulmonary / or exp Lung

#4 #1 and #2 and #3

#5 exp surgical procedures operative/ or exp Thoracic surgery, Video-Assisted / or exp Microsurgery/or exp Surgical Procedures Minimally Invasive/ or exp Robotics/

#6 (surgery or surgical*).mp.

#7 (Video-Assisted* or Video Assisted* or Video* or Thorac* or VATS).mp.

For peer review only

#8 (robot*).mp.

#9(computer guid* or computer-guid* or computer-assisted or computer assisted).mp.

#10(da Vinci or Zeus or telesurgery).mp.

#11 #5or #6 or #7 or #8 or #9 or #10

#12 randomized controlled trial.pt.

#13 controlled clinical trial.pt.

#14 randomized.ab.

#15 placebo.ab.

#16 clinical trials as topic.sh.

#17 randomly.ab.

#18 trial.ti.

#19 #12 or #13 or #14 or #15 or #16 or #17 or #18

#20 (animals not (humans and animals)).sh.

#21 #19 not #20

#22 #4 and #11 and #21

Search strategy for Embase as follows:

#1 exp neuromuscular blockade/

#2 neuromusc*.mp.

#3 exp muscle relaxation/

#4 (Deep or profound or intense or extreme or depth).mp.

#5 exp Pulmonary /

#6 exp Lung/

#7 exp Surgical Procedures Operative/

#8 exp Thoracic surgery, Video-Assisted /

#9 exp Microsurgery/

#10 exp Surgical Procedures Minimally Invasive/

#11 exp Robotic/

#12 (surgery or surgical*).mp.

For peer review only

#13 (robot*).mp.

#14(Video-Assisted* or Video Assisted* or Video* or Thorac* or VATS).mp.

#15 (computer guid* or computer-guid* or computer-assisted or computer assisted).mp.

#16 (da Vinci or Zeus or telesurgery).mp.

#17 Clinical trial.mp.

#18 (placebo*).mp.

#19 exp randomized controlled trial/

#20 (random*).mp.

#21 (exp animal/ or nonhuman/ or exp animal experiment/) not human/

#22 #1 or #2 or #3

#23 #22 and #4

#24 #5 or #6

#25 #7 or #8 or #9 or #10 or #11 or #12 or #13 or #14 or #15 or #16

#26 #23 and #24 and #25

#27 #17 or #18 or #19 or #20

#28 #26 and #27

#29 #28 not #21

For peer review only

PRISMA-P checklist

Table PRISMA-P (preferred reporting items for systematic review and meta-analysis protocols) 2015 checklist: recommended items to address in a systematic review protocol

Section and topic	Item No	Checklist item	Reported on page #
**Administrative information**			
Title:			
Identification	1a	Identify the report as a protocol of a systematic review	1
Update	1b	If the protocol is for an update of a previous systematic review, identify as such	None
Registration	2	If registered, provide the name of the registry (such as PROSPERO) and registration number	3,5
Authors:			
Contact	3a	Provide name, institutional affiliation, e-mail address of all protocol authors; provide physical mailing address of corresponding author	1
Contributions	3b	Describe contributions of protocol authors and identify the guarantor of the review	20
Amendments	4	If the protocol represents an amendment of a previously completed or published protocol, identify as such and list changes; otherwise, state plan for documenting important protocol amendments	None
Support:			
Sponsor	5b	Provide name for the review funder and/or sponsor	None
Role of sponsor or funder	5c	Describe roles of funder(s), sponsor(s), and/or institution(s), if any, in developing the protocol	None
**Introduction**			
Rationale	6	Describe the rationale for the review in the context of what is already known	4-6
Objectives	7	Provide an explicit statement of the question(s) the review will address with reference to participants, interventions, comparators, and outcomes (PICO)	6
**Methods**			
Eligibility criteria	8	Specify the study characteristics (such as PICO, study design, setting, time frame) and report characteristics (such as years considered, language, publication status) to be used as criteria for eligibility for the review	6-9

Information sources	9	Describe all intended information sources (such as electronic databases, contact with study authors, trial registers or other grey literature sources) with planned dates of coverage	9-11
Search strategy	10	Present draft of search strategy to be used for at least one electronic database, including planned limits, such that it could be repeated	10-11, S1
Study records:			
Data management	11a	Describe the mechanism(s) that will be used to manage records and data throughout the review	11
Selection process	11b	State the process that will be used for selecting studies (such as two independent reviewers) through each phase of the review (that is, screening, eligibility and inclusion in meta-analysis)	12
Data collection process	11c	Describe planned method of extracting data from reports (such as piloting forms, done independently, in duplicate), any processes for obtaining and confirming data from investigators	12-13
Data items	12	List and define all variables for which data will be sought (such as PICO items, funding sources), any pre-planned data assumptions and simplifications	11-12
Outcomes and prioritization	13	List and define all outcomes for which data will be sought, including prioritization of main and additional outcomes, with rationale	8-9
Risk of bias in individual studies	14	Describe anticipated methods for assessing risk of bias of individual studies, including whether this will be done at the outcome or study level, or both; state how this information will be used in data synthesis	14-15
Data synthesis	15a	Describe criteria under which study data will be quantitatively synthesised	16
	15b	If data are appropriate for quantitative synthesis, describe planned summary measures, methods of handling data and methods of combining data from studies, including any planned exploration of consistency (such as I^2, Kendall's τ)	16
	15c	Describe any proposed additional analyses (such as sensitivity or subgroup analyses, meta-regression)	16-18
	15d	If quantitative synthesis is not appropriate, describe the type of summary planned	16
Meta-bias(es)	16	Specify any planned assessment of meta-bias(es) (such as publication bias across studies, selective reporting within studies)	18
Confidence in cumulative evidence	17	Describe how the strength of the body of evidence will be assessed (such as GRADE)	14-15;16-17

Deep neuromuscular block for minimally invasive lung surgery: a protocol for a systematic review with meta-analysis and trial sequential analysis

Journal:	*BMJ Open*
Manuscript ID	bmjopen-2021-056816.R1
Article Type:	Protocol
Date Submitted by the Author:	13-Feb-2022
Complete List of Authors:	Zheng, Jianqiao; Sichuan University West China Hospital, Department of Anesthesiology<br>Du, Li; University of Electronic Science and Technology of China, Department of Anesthesiology<br>Deng, Xiaoqian; Sichuan University West China Hospital, Department of Anesthesiology<br>Zhang, Lu; Sichuan University West China Hospital, Department of Anesthesiology<br>Wang, Jia; Sichuan University West China Hospital, Department of Anesthesiology<br>Chen, Guo; Sichuan University West China Hospital, Department of Anesthesiology
**Primary Subject Heading**:	Surgery
Secondary Subject Heading:	Anaesthesia, Surgery
Keywords:	Cardiothoracic surgery < SURGERY, Adult anaesthesia < ANAESTHETICS, Adult surgery < SURGERY

SCHOLARONE™
Manuscripts

TITLE PAGE

Deep neuromuscular block for minimally invasive lung surgery: a protocol for a systematic review with meta-analysis and trial sequential analysis

Authors

Jianqiao Zheng[1] E-mail: zhjq1983@163.com

Li Du[2] E-mail: huaying-duli@163.com

Xiaoqian Deng[1] E-mail: 50058837@qq.com

Lu Zhang[1] E-mail: 304022514@qq.com

Jia Wang[1] E-mail: 19983157894@qq.com

*Guo Chen[1] E-mail: Anesthesiology_SCU@163.com

[1]Department of Anesthesiology, West China Hospital, Sichuan University No. 37th, Guoxue Alley, Wuhou District, Chengdu 610041, Sichuan, China.

[2]Department of Anesthesiology, Sichuan Cancer Hospital & Institute, Sichuan Cancer Center, School of Medicine, University of Electronic Science and Technology of China, No.55th, People's South Road, Chengdu 610041, Sichuan, China.

*Corresponding Author

Name: Guo Chen

E-mail: Anesthesiology_SCU@163.com

Phone: +86-28-8542-3593

Address: Department of Anesthesiology, Sichuan University West China Hospital, No. 37th, Guoxue Alley, Wuhou District, Chengdu 610041, Sichuan, China.

Words: 3526

For peer review only

ABSTRACT

Introduction Minimally invasive lung surgery (MILS) gradually became the primary surgical therapy for lung cancer, which remains the leading cause of cancer death. Adequate muscle relaxation by deep neuromuscular block (NMB) is particularly necessary for MILS to provide a satisfactory surgical field. But deep NMB for MILS remains controversial, as one-lung ventilation may provide an acceptable surgical field. Then, we will perform a protocol for a systematic review and meta-analysis to identify the efficacy of deep NMB for MILS.

Methods and analysis We will search the PubMed, Web of Science, Cochrane Library, Ovid Medline, Embase, China National Knowledge Infrastructure, Chinese BioMedical Literature, Wanfang and VIP databases from inception to March 2022 to identify randomized controlled trials of adult participants undergoing MILS with deep NMB. Studies published in English or Chinese will be considered. The primary outcome will be the surgical conditions according to the surgeon's perspective. Secondary outcomes will be the incidence of perioperative events and perioperative mortality. Heterogeneity will be assessed by the χ^2 test and I^2 statistic. Data will be synthesized by either fixed-effects or random-effects models according to the I^2 value. Cochrane risk-of-bias tool, trial sequential analysis and GRADE (Grading of Recommendations Assessment, Development and Evaluation) will be used to assess the evidence quality

For peer review only - http://bmjopen.bmj.com/site/about/guidelines.xhtml

and control the risks of random errors. Funnel plots and Egger's regression test will be used to assess publication bias.

Ethics and dissemination Ethical approval was not required for this systematic review protocol. The findings will be disseminated through peer-reviewed publications.

Keywords deep neuromuscular block, minimally invasive, thoracoscopic, pulmonary, meta-analysis, randomized controlled trial.

PROSPERO registration number CRD42021254016

Strengths and limitations of the study

► This systematic review protocol follows the Preferred Reporting Items for Systematic Review and Meta-Analysis Protocols (PRISMA-P) guidelines to conduct a rigorous risk of bias assessment.

► Trial sequential analysis will be used to control the risks of false positives by calculating the diversity adjusted information size for the outcomes.

► Funnel plots and Egger's regression test will be used to assess publication bias.

► Subgroup analysis will be performed to assess the heterogeneity according to patients' age, body mass index, and type of MILS.

INTRODUCTION

Lung cancer remains the leading cause of cancer death, with an estimated 1.8 million new deaths in 2020, accounting for 18% of the total

For peer review only - http://bmjopen.bmj.com/site/about/guidelines.xhtml

cancer deaths according to Global Cancer Statistics 2020. [1] Lung cancer is the second most common cancer, with an estimated 2.2 million new cases in 2020, representing 11.4% of all cancer cases. [1] Due to its the high incidence and mortality, the treatment of lung cancer is a global challenge.

Surgical resection remains the primary therapy in the treatment of lung cancer. Since the 1990s, minimally invasive surgical techniques of video-assisted thoracic surgery and robotic-assisted thoracic surgery have been applied in the diagnosis and treatment of intrathoracic diseases. [2-4] Growing experience with minimally invasive lung surgery (MILS), combined with improvements in video technology and instrumentation, has allowed conventional thoracotomy to be gradually replaced by MILS in recent years.[5-8]

Recent literature suggests that MILS was equivalent to open thoracotomy on long-term survival and overall oncologic efficacy, even with a better short-term survival.[9-15] The minimally invasive surgical approach is still the favored surgical procedure in that it offers many advantages, including less trauma and pain, faster recovery, fewer complications, lower immunological responses, and a shorter hospitalization period. [16-21] In addition, it is associated with a higher tolerance to postoperative adjuvant therapy and mitigates or ameliorates the postoperative decline of health-related functional status. [22-25]

Adequate muscle relaxation by deep neuromuscular block (NMB) is

For peer review only - http://bmjopen.bmj.com/site/about/guidelines.xhtml

particularly necessary for minimally invasive surgical techniques.[26-28] MILS involves areas adjacent to major blood vessels and can trigger intraoperative body movement, cough, and diaphragm movement.[29] Moreover, the diaphragm is the most resistant muscle to neuromuscular blocking agents (NMBAs), and movement of the diaphragm can interfere with the surgical procedure. Deep NMB can inhibit the response to carinal stimulation and prevent bucking and coughing during surgical procedures. [30-32] In addition, it can reduce the peak pressure and plateau pressure and improve lung compliance and peripheral oxygen saturation during one-lung ventilation. [33]

There is still controversy regarding the clinical benefit of maintaining deep NMB for MILS because deep NMB seems unnecessary, as ribcage provides thoracic support and one-lung ventilation usually provides a satisfactory surgical field. In addition, the risk of residual neuromuscular block is estimated to occur in 26% to 88% of patients undergoing general anesthesia, and this incidence is inevitably increased after deep NMB. [34 35] Numerous clinical studies have documented that postoperative residual neuromuscular block has the potential risk of increasing the incidence of postoperative pulmonary complications (such as airway obstruction, aspiration, and hypoxia), the odds of hospital readmission intensive care unit admission and the hospital length of stay. [36-40]

Hence, the clinical benefits of deep NMB for MILS remain

For peer review only - http://bmjopen.bmj.com/site/about/guidelines.xhtml

controversial. Therefore, it is necessary to conduct a systematic review and meta-analysis to analyse the clinical efficacy of deep NMB on MILS. The outcomes of this systematic review will provide evidence for better clinical decision making and possible directions for further clinical trials.

Objectives

We are conducting this protocol of systematic review and meta-analysis to determine the clinical efficacy of deep NMB on surgical conditions of MILS according to the surgeon’s perspective. Patients' postoperative recovery and the incidence of perioperative events will also be identified. Furthermore, we will use trial sequential analysis (TSA) to confirm the reliability of the results.

METHODS AND ANALYSIS

Study design

Our review protocol has been registered with PROSPERO (registration number: CRD 42021254016). This protocol was prepared according to the Preferred Reporting Items for Systematic Reviews and Meta-Analyses Protocols (PRISMA-P) guidelines.[41] The systematic review and meta-analysis will be conducted in accordance with the Cochrane Handbook and reported according to the PRISMA statement.[42,43] The study is expected to begin searching in March 2022 and complete in May 2022.

Inclusion/exclusion criteria for study selection

For peer review only - http://bmjopen.bmj.com/site/about/guidelines.xhtml

Types of studies

We will include all randomized controlled trials (RCTs) that evaluated deep NMB for MILS. Only studies published in English or Chinese will be included.

Studies with the following situations will be excluded: (1) studies without control group, compared deep NMB produced by different kinds of NMBAs only; (2) studies with incomplete, incorrect data or research data that could not be used for statistical analysis; and (3) studies that were abstracts from conferences, editorials, duplicate publications, letters, reviews, observational studies, and retrospective studies.

Types of participants

Adult participants (age ≥18 years old) undergoing any kind of MILS (including thoracoscopic surgery, video-assisted thoracic surgery or robotic-assisted thoracic surgery) with deep NMB will be included. There will be no limits on study participants in terms of gender, ethnicity, and body mass index (BMI).

Types of interventions/controls

In the intervention group, participants had to receive deep NMB [defined as a train-of-four (TOF) count of zero or a post-tetanic count (PTC) ≥1] or intense (profound) NMB [defined as a train-of-four (TOF) count =0 or a post-tetanic count (PTC) =0] throughout the MILS.[44]

The control group will be the participants who received shallow NMB

For peer review only - http://bmjopen.bmj.com/site/about/guidelines.xhtml

(defined as a TOF count =4 or Measured TOF Ratio= 0.1-0.4), moderate NMB (defined as TOF count=1-3) or without NMBAs throughout the MILS.[44]

Types of outcome measures

Meta-analysis is not possible with no studies, or only one study. So, all the outcomes will be reported only if reported by at least 2 RCTs.

Primary outcomes

The primary outcome will be the surgical conditions of the MILS according to the surgeon's perspective. Surgical conditions were evaluated as a surgical rating scale or the percentage of patients with clinically acceptable surgical conditions (Clinically acceptable surgical conditions were defined as Acceptable, Good or Optimal conditions) (table 1).[45]

Table 1 Surgical rating scale (SRS)

SRS category (scale)	Conditions Description
Extremely poor conditions (Score 1)	The surgeon is unable to work because of coughing or of the inability to obtain a visible field because of inadequate muscle relaxation.
Poor conditions (Score 2)	There is a visible field, but the surgeon is severely hampered by inadequate muscle relaxation with continuous muscle contractions, movements, or both.
Acceptable conditions (Score 3)	There is a wide visible field but muscle contractions, movements, or both occur regularly
Good conditions (Score 4)	A wide working field with sporadic muscle contractions, movements, or both
Optimal conditions (Score 5)	A wide visible working field without any movement or contractions.

Secondary outcomes

1. The incidence of perioperative events included the following:

►Incidence of intraoperative events: defined as body movement, coughing, and breathing against the ventilator (with the aid of airway pressure

For peer review only - http://bmjopen.bmj.com/site/about/guidelines.xhtml

monitoring and capnography).

►Incidence of postoperative pulmonary complications: defined as the composite of any respiratory infection, respiratory failure, pleural effusion, atelectasis, or pneumothorax.

2. Perioperative mortality

►defined as all-cause death during operation procedure, within 30 days after surgery, or death during hospitalization.

3. Patients' postoperative recovery

►Recovery time of NMB: defined as the time from administration of the reversal agent to the achievement of a TOF ratio of 0.9.

4. Duration of surgery

Search strategy

We will search English and Chinese electronic databases from inception to March 2022 for published literature. The English databases included PubMed, Web of Science, Cochrane Library, Ovid Medline and Embase. The Chinese databases included China National Knowledge Infrastructure (CNKI), Chinese BioMedical Literature (CBM), Wanfang database and VIP Database. We will also scrutinize the reference lists of each study and trial registry database (WHO International Clinical Trials Registry Platform and Clinical Trials.gov) for missing studies and unpublished or ongoing clinical trials. After data extraction, we will ask the corresponding authors of the included literature for more grey literature

For peer review only

For peer review only - http://bmjopen.bmj.com/site/about/guidelines.xhtml

to avoid potential missing data as much as possible.

The search strategy for PubMed (as an example) is shown in table 2. The following search terms will be used: deep neuromuscular block, minimally invasive, thoracoscopic, video assisted, robotic assisted, pulmonary, and randomized controlled trial. The search terms will be translated into Chinese for study identification in Chinese databases. We will perform a new search in the databases to check if any studies were published during the elaboration of the systematic review before the final publication. The preliminary search strategy is given in (online supplementary additional file 1).

Table 2 Search strategy for PubMed

No	Search terms
#1	"Neuromuscular blockade"[MeSH] OR neuromusc*[tiab] OR "muscle relaxation" [MeSH]
#2	Deep[tiab] OR profound[tiab] OR intense[tiab] OR extreme[tiab] OR depth[tiab]
#3	"Pulmonary" [Mesh] OR "Lung" [Mesh] OR Pulmonary [tiab] OR Lung [tiab]
#4	"Surgical Procedures Operative" [Mesh] OR "Microsurgery" [Mesh] OR "Surgical Procedures Minimally Invasive" [Mesh] OR Minimally Invasive Surgery[tiab] OR MIS [tiab] OR Minimal Access Surgical Procedures [tiab]OR Minimal Surgical Procedures[tiab] OR Minimally Invasive Surgical Procedures [tiab] OR Minimal Surgical Procedure[tiab] OR minimally invasive surgical procedure [tiab] OR minimal access surgical procedure[tiab]
#5	"Thoracic surgery, Video-Assisted" [Mesh] or Surgeries, Video-Assisted Thoracic [af] or Surgery, Video-Assisted Thoracic [af] or Thoracic Surgeries, Video-Assisted [af] or Thoracic surgery, Video-Assisted [af] or Video-Assisted Thoracic Surgeries [af] or Video-Assisted Thoracic Surgery [af] or Surgeries, Video-Assisted Thoracoscopic [af] or Surgery, Video-Assisted Thoracoscopic [af] or Thoracoscopic Surgeries, Video-Assisted [af] or Thoracoscopic Surgery, Video-Assisted [af] or Video Assisted Thoracoscopic Surgery [af] or Video Assisted Thoracoscopic Surgeries [af] or Video-Assisted Thoracic Surgery [af] or Video Assisted Thoracic

For peer review only - http://bmjopen.bmj.com/site/about/guidelines.xhtml

	Surgery [af] or Surgery, Thoracic, Video-Assisted [af] or VATS [af] or VATSs [af].
#6	"Robotics" [MeSH] OR robot* [tiab] OR computer guid*[tiab] OR computer-guid*[tiab] OR computer-assisted[tiab] OR computer assisted [tiab]OR da Vinci [tiab]OR Zeus [tiab]OR telesurgery[tiab]
#7	#1 AND #2 AND #3
#8	#4 OR #5 OR #6
#9	"Controlled clinical trial" [Publication Type] OR "randomized controlled trial" [Publication Type] OR "randomized" [Title/Abstract] OR "randomized" [Title/Abstract] OR "Placebo" [Title/Abstract] OR "randomly" [Title/Abstract] OR "Clinical trial" [Title]
#10	"animals" [MeSH] NOT ("human" [MeSH] AND "animals" [MeSH])
#11	#7and #8 and #9 not #10

Data collection and analysis

Selection of studies

Two reviewers (Z-JQ and WJ) will be required to independently screen the retrieved studies. Briefly, they will exclude duplicate studies and those not matching the inclusion criteria by reading the titles and abstracts. After reading the full text of each study, studies meeting the inclusion criteria will be selected. Any disagreements will be resolved through discussion with a third reviewer (DL). A fourth reviewer (CC) will check all procedures before approving the data extraction. Details of the entire study selection procedure will be shown in the PRISMA flow diagram (figure 1).

Data extraction

Two reviewers (Z-JQ and ZL) will extract data from the included studies independently following data acquisition with Microsoft Excel software. Required information including demographic data, type of MILS,

For peer review only - http://bmjopen.bmj.com/site/about/guidelines.xhtml

inclusion/exclusion criteria, level of NMB during MILS (definition and measurement), outcome indicators (primary, secondary, and exploratory outcomes), etc. Information about study design (such as randomization, allocation concealment, blinding methods, data collection and statistical analysis, outcome reporting) will also be recorded for the next step quality assessment. The resulting data will be recorded as the mean± SD for continuous variables, and the proportion of participants with percentages for dichotomous data. If necessary, a third reviewer (D-XQ) will double-check the data to ensure consistency. If information and data are missing or incomplete in any study, we will contact with the corresponding authors of the literature to obtain the original data by email. If necessary, we will extract numerical data from graphs using Adobe Photoshop as described by Gheibi et al.[46] A detailed list of the information and data to be extracted is presented in table 3.

Table 3 Data and information extraction schedule

Subject	Content
Publication information	Name of the first author; contact email; publish year; country; corporate sponsorship.
Participant	Sample size; Age; Sex; Height and weight or body mass index (BMI); American Society of Anesthesiologists (ASA) physical status classification levels; Type of MILS; Inclusion and exclusion criteria if necessary.
Intervention	Level of NMB (deep NMB, intense NMB or profound NMB); Assessment of the NMB level (equipment of neuromuscular function monitor; monitor position); Type of neuromuscular blocking agents (NMBAs); Dose and administration of NMBAs; Administration of NMBAs antagonist (sugammadex or neostigmine).
Control	Level of NMB (moderate NMB; shallow NMB or without NMBAs); Assessment of the DNMB (equipment for neuromuscular function monitor; monitor position); Type of neuromuscular blocking agents (NMBAs); Dose and administration of NMBAs; Administration of NMBAs antagonist (sugammadex or neostigmine).

For peer review only - http://bmjopen.bmj.com/site/about/guidelines.xhtml

Outcome	Primary outcome (Surgical rating scale or the percentage of patients with clinically acceptable surgical conditions); Secondary outcome measurements (Perioperative events; Perioperative mortality; Patients' postoperative recovery; Duration of surgery).
Study design	Application of randomization and blinding; Description about allocation concealment; Statistical analysis; Sample size calculation; Outcome reporting.
Other information	Intraoperative temperatures; Bispectral index (BIS) values; Time or condition of tracheal intubation and extubation; Type of anesthesia maintenance technique (Inhalation anesthesia; Total intravenous anesthesia; or both); Duration of anesthesia.

Quality assessment

Two reviewers (WJ and ZL) will independently assess the risk of bias in the included studies with the guidance of the Cochrane risk of bias tool.[47] We will evaluate the methodology with respect to random sequence generation, allocation concealment, blinding of participants and personnel, blinding of outcome assessment, incomplete outcome data, selective outcome reporting, other risks of bias and overall risk of bias. The risk of bias components will be scored as three levels (low risk, unclear and high risk) in accordance with the item in the checklist. If all risk of bias domains were scored as having a low risk of bias, the trial will be defined as having a low overall risk of bias. If one or more of the bias domains were scored as unclear risk of bias or high risk of bias, the trial will be defined as having a high overall risk of bias. Trials with a low risk of bias in all domains of sequence generation, allocation concealment, blinding, incomplete data, selective outcome reporting, and other risks of bias will be classified as having an overall low risk of bias. Trials with one or more of these domains scored as high or unclear risk of bias will be classified as having an overall

For peer review only - http://bmjopen.bmj.com/site/about/guidelines.xhtml

high risk of bias.[48 49] If any disagreements, the risk assignment will be settled through arbitration of a third reviewer (GC). Classification of the trials will follow criteria defined in online supplemental additional file 2.

Measures of treatment effect

For continuous outcome data, the mean differences (MDs) (outcome data reported by same scale) or the standardized mean difference (SMDs) (outcome data reported by different scales) with 95% confidence intervals (CIs) will be used for analysis. For dichotomous data, the relative risks (RR) with 95% CIs will be used for analysis.

Assessment of heterogeneity

The choice between a fixed-effect and a random-effects meta-analysis based on statistical heterogeneity is not recommended by Cochrane guidelines.[42] In order to testify the result by the traditional meta-analysis method based on statistical heterogeneity (Statistical heterogeneity will be assessed by the standard χ^2 test and I^2 test. If $p \geq 0.1$ and if $I^2 \leq 50\%$, a fixed-effects model will be used. If $p < 0.1$ or $I^2 > 50\%$, random-effects models will be applied), a pragmatic approach will be performed to undertake both a fixed-effect and a random-effects meta-analysis for each outcome, with an intention to present the random-effects result if there is no indication of funnel plot asymmetry.[42] If there is an indication of funnel plot asymmetry, then both methods are problematic. It may be reasonable to present both analyses or neither, or to perform a sensitivity analysis in which small

For peer review only - http://bmjopen.bmj.com/site/about/guidelines.xhtml

studies are excluded or addressed directly using meta-regression. A P value <0.05 was assumed as statistically significant.

Trial Sequential Analysis

Trial sequential analysis (TSA) will be performed by Stata/MP 16.0 (Stata Corp, College Station, TX, USA) to control the risks of false positives by calculating the required information size (RIS). [50-52] RIS is the number of participants needed in a meta-analysis to detect or reject a certain intervention effect. [53 54] RIS and information size for each outcome will be calculated. In addition, the cumulative Z-curve's breach of relevant trial sequential to monitor boundaries will be calculated for all outcomes.[53 54]

For continuous outcomes, we will use the observed SD, a mean difference of the observed SD/2, an alpha of 2.5% and a beta of 10% for primary and secondary outcomes in the TSA.[55] For dichotomous outcomes, we will use the proportion of participants with an outcome in the control group, a relative risk reduction of 0.20, and an alpha of 0.025 and a beta of 0.10 in the TSA.[56]TSA will be performed using the TSA program version 0.9.5.10 Beta (http://www.ctu.dk/tsa).[57]

Diversity adjusted information size (*DIS*) should be calculated, as the required information size might be underestimated. We will use the formula: DIS= $SS/1- D^2$ (D^2: Diversity, is the percentage of the variability between trials to the within-trial variance and constitutes the percentage of

For peer review only - http://bmjopen.bmj.com/site/about/guidelines.xhtml

the variability between trials to the total variance in the meta-analysis. *SS*: Sample size in a single randomized clinical trial).[58]

Subgroup analysis

We will further explain the results with an analysis of subgroups or subsets. If sufficient trials are available (the subgroup analysis will be performed if the variable is reported by at least 2 RCTs), data from different age, different body mass indexes (BMIs) and different types of MILSs will be analysed separately.

► Different ages (deep NMB for MILS in patients with different ages as follows: 18 years≤ Patients<65 years; 65 years≤ Patients<75 years; Patients≥75 years).

► Different types of MILS (deep NMB for video-assisted thoracoscopic lung surgery; deep NMB for robotic-assisted thoracoscopic lung surgery).

► Different BMIs (deep NMB for MILS in patients with different BMIs as follows: BMI<25.0 kg/m^2; 25.0 kg/m^2 ≤ BMI < 30 kg/m^2; BMI ≥ 30kg/m^2).

To determine whether a statistically significant subgroup difference was detected, the p value from the test for subgroup differences will be considered. If a significant difference between subgroups is identified (test for interaction $p<0.05$), we will report the results for individual subgroups separately.[42]

Sensitivity analysis

For peer review only - http://bmjopen.bmj.com/site/about/guidelines.xhtml

After analysis of subgroups or subsets, sensitivity analysis will be used to evaluate how uncertain assumptions of data and usage affect the robustness of the combined results. We will exclude low quality studies (defined as high risk of bias studies according to the Cochrane risk of bias tool assessment), reanalyse the included studies, and assess whether there are significant differences between the combined effects. If necessary, we will remove the included studies one by one to observe whether the pooled estimations are stable. Significant changes may indicate significant heterogeneity among studies.

Assessment of publication biases

The potential publication bias will be statistically analysed using funnel plot analysis and Egger's regression test, while no less than 10 original studies are involved for an outcome.[59 60] The trim-and-fill analysis will also be performed to adjust any potential publication bias, as it is based on the assumption that the effect sizes of all the studies are normally distributed around the center of a funnel plot in the absence of publication bias.[61] Publication biases will be performed by Stata/MP 16.0 (Stata Corp, College Station, TX, USA).

Grading the quality of evidence

The quality of evidence for all the outcomes will be assessed using the GRADE approach through risk of bias, consistency, objectivity, accuracy and reported bias. [62] The certainty of evidence will be classified

For peer review only - http://bmjopen.bmj.com/site/about/guidelines.xhtml

as high, moderate, low or very low. According to GRADE, data from randomized controlled trials are considered high quality evidence but can be rated down according to risk of bias, imprecision, inconsistency, indirectness or publication bias.

Patient and public involvement statement

Patients or the public were not involved in the design, conduct, reporting, or dissemination plans of our research.

DISCUSSION

This systematic review will provide an overview of the current state of evidence concerning the clinical efficacy of deep NMB for MILS. We will examine the effect of deep NMB on surgical conditions according to the surgeon's perspective. In addition, we will investigate the efficacy of deep NMB on patients' postoperative recovery and postoperative complications. To the best of our knowledge, this will be the first systematic review concerning this topic. The outcomes of this systematic review will provide evidence for better clinical decision making on the management of NMB and patient care during MILS.

This systematic review protocol follows the Preferred Reporting Items for Systematic Review and Meta-Analyses Protocols (PRISMA-P) guidelines. The strengths of our systematic review include the following: First, we performed a comprehensive search of English and Chinese databases. Second, multivariable analysis (including assessment of study

For peer review only - http://bmjopen.bmj.com/site/about/guidelines.xhtml

quality, subgroup analysis, sensitivity analysis, trial sequential analysis and Egger's regression test) will be used to minimize the confounding bias. Third, screening, data extraction and quality assessment will be performed by two independent reviewers according to the guidelines.

There are also limitations to our analysis. First, studies with different NMBA and NMBA antagonists (sugammadex or neostigmine) will be included, resulting in potential heterogeneity. Second, the number of studies with eligible data for subgroup analyses may be limited. Third, the sample size in each study may be small. Fourth, another limitation may be the current lack of high-level evidence, such as well-designed randomized controlled trials with double-blind designs. Thus, we will use rigorous methods such as TSA and trim-and-fill analysis in the data analysis and will meta-analyse the outcomes as appropriate.

ETHICS AND DISSEMINATION

Ethical approval was not required for this systematic review protocol. The findings will be disseminated through peer-reviewed publications.

Timelines

Formal screening of search results will begin in March 2022. Data extraction will begin in April 2022. The project is due to complete in May 2022.

Author Contributors

Z-JQ and DL conceived the idea for this systematic review. All

For peer review only

authors (Z-JQ, DL, WJ, ZL, D-XQ, CC) developed the methodology for the systematic review. The manuscript was drafted by Z-JQ and DL, and revised by all authors. D-XQ and CC will screen potential studies, and perform duplicate independent data abstraction. Z-JQ and ZL will undertake risk of bias assessment and assess the evidence quality. Z-JQ and DL will conduct the data synthesis. All authors contributed to the research and agreed to be responsible for all aspects of the work.

Funding

None.

Competing interests

None declared.

Data availability statement

Not applicable for this protocol.

Patient consent for publication

No patient was involved.

Provenance and peer review

Not commissioned; externally peer reviewed.

REFERENCES

1 Sung H, Ferlay J, Siegel RL, *et al*. Global Cancer Statistics 2020: GLOBOCAN Estimates of Incidence and Mortality Worldwide for 36 Cancers in 185 Countries. *CA Cancer J Clin* 2021; 71:209-49.

2 McKenna RJ Jr, Houck W, Fuller CB. Video-assisted thoracic surgery lobectomy: experience with 1,100 cases. *Ann Thorac Surg* 2006;81: 421-5; discussion 425-6.

3 Fahim C, Hanna W, Waddell T, *et al*. Robotic-assisted thoracoscopic surgery for lung resection: the first Canadian series. *Can J Surg*2017; 60:260-65.

4 Ujiie H, Gregor A, Yasufuku K. Minimally invasive surgical approaches for lung cancer.

For peer review only - http://bmjopen.bmj.com/site/about/guidelines.xhtml

Expert Rev Respir Med 2019;13(6):571-78.

5 Sihoe AD. Uniportal video-assisted thoracic (VATS) lobectomy. Ann *Cardiothorac Surg* 2016; 5:133-44.

6 Hernandez-Arenas LA, Lin L, Yang Y, *et al*. Initial experience in uniportal subxiphoid video-assisted thoracoscopic surgery for major lung resections. *Eur J Cardiothorac Surg* 2016;50: 1060-66.

7 Ujiie H, Yasufuku K. New era of "resection of the carina and lower trachea. *J Thorac Dis* 2017; 9:4932-36.

8 Ujiie H, Kato T, Hu HP, *et al*. A novel minimally invasive near-infrared thoracoscopic localization technique of small pulmonary nodules: A phase I feasibility trial. *J Thorac Cardiovasc Surg* 2017; 154:702-11.

9 Boffa DJ, Kosinski AS, Furnary AP, *et al*. Minimally Invasive Lung Cancer Surgery Performed by Thoracic Surgeons as Effective as Thoracotomy. *J Clin Oncol* 2018; 36:2378-85.

10 Long H, Tan Q, Luo Q, *et al*. Thoracoscopic surgery versus thoracotomy for lung cancer: short-term outcomes of a randomized trial. *Ann Thorac Surg* 2018; 105:386-92.

11 Weksler B, Tavares J, Newhook TE, *et al*. Robot-assisted thymectomy is superior to transsternal thymectomy. *Surg Endosc* 2012; 26:261-6.

12 Han KN, Kim HK, Choi YH. Midterm outcomes of single port thoracoscopic surgery for major pulmonary resection. *PloS one* 2017;12: e0186857.

13 Shen H, Wang X, Nie Y, *et al*. Minimally invasive surgery versus thoracotomy for resectable stage II and III non-small-cell lung cancers: a systematic review and meta-analysis. *Eur J Cardiothorac Surg* 2020; 59: 940-50.

14 O'Sullivan KE, Kreaden US, Hebert AE, *et al*. A systematic review and meta-analysis of robotic versus open and video-assisted thoracoscopic surgery approaches for lobectomy. *Interact Cardiovasc Thorac Surg* 2019; 28:526-34.

15 Yang CJ, Kumar A, Klapper JA, *et al*. A national analysis of long-term survival following thoracoscopic versus open lobectomy for stage I non-small-cell lung cancer. *Annals of surgery* 2019; 269:163-171.

For peer review only - http://bmjopen.bmj.com/site/about/guidelines.xhtml

16 O'Sullivan KE, Kreaden US, Hebert AE, *et al*. A systematic review and meta-analysis of robotic versus open and video-assisted thoracoscopic surgery approaches for lobectomy. *Interact Cardiovasc Thorac Surg* 2019; 28:526-34.

17 Li Z, Xia M, Liu C, *et al*. A meta-analysis of minimally invasive surgery versus thoracotomy for centrally located non-small cell lung cancer. *J Thorac Dis* 2021; 13:252-61.

18 Bendixen M, Jørgensen OD, Kronborg C, *et al*. Postoperative pain and quality of life after lobectomy via video-assisted thoracoscopic surgery or anterolateral thoracotomy for early stage lung cancer: a randomised controlled trial. *Lancet Oncol* 2016; 17: 836-44.

19 Swanson SJ, Miller DL, McKenna RJ Jr., et al. Comparing robot-assisted thoracic surgical lobectomy with conventional video-assisted thoracic surgical lobectomy and wedge resection: results from a multihospital database (Premier). *J Thorac Cardiovasc Surg* 2014;147: 929-37.

20 Paul S, Jalbert J, Isaacs AJ, et al. Comparative effectiveness of robotic-assisted vs thoracoscopic lobectomy. *Chest* 2014;146: 1505-12.

21 Guerrera F, Olland A, Ruffini E, *et al.* VATS lobectomy vs. open lobectomy for early-stage lung cancer: an endless question-are we close to a definite answer? *J Thorac Dis* 2019; 11:5616-8.

22 Swanson SJ, Herndon JE, D'Amico TA, et al. Video- assisted thoracic surgery lobectomy: report of CALGB 39802-a prospective, multi-institution feasibility study. *J Clin Oncol* 2007; 25:4993-7.

23 Cao C, Manganas C, Ang SC, *et al*. Video-assisted thoracic surgery versus open thoracotomy for non-small cell lung cancer: a meta-analysis of propensity score-matched patients. *Interact Cardiovasc Thorac Surg* 2013; 16:244-9.

24 Guerrera F, Olland A, Ruffini E, *et al*. VATS lobectomy vs. open lobectomy for early-stage lung cancer: an endless question-are we close to a definite answer? *J Thorac Dis* 2019;11: 5616-8.

25 Handy JR Jr. Minimally invasive lung surgery and postoperative quality of life. *Thorac Surg Clin* 2012;22:487-95.

For peer review only - http://bmjopen.bmj.com/site/about/guidelines.xhtml

26 Iwata H. Minimally invasive pulmonary surgery for lung cancer, up to date. *Gen Thorac Cardiovasc Surg* 2013; 61:449–54.

27 Barrio J, Errando CL, Miguel GS, *et al*. Effect of depth of neuromuscular blockade on the abdominal space during pneumoperitoneum establishment in laparoscopic surgery. *J Clin Anesth* 2016; 34:197-203.

28 Kim HJ, Lee K, Park WK, *et al*. Deep neuromuscular block improves the surgical conditions for laryngeal microsurgery. *Br J Anaesth* 2015; 115:867-72.

29 Lesser TG, Schubert H, Güllmar D, *et al*. One-lung flooding reduces the ipsilateral diaphragm motion during mechanical ventilation. *Eur J Med Res* 2016; 21:9.

30 Gibson PG, Simpson JL, Ryan NM, *et al*. Mechanisms of cough. *Curr Opin Allergy Clin Immunol* 2014; 14:55-61.

31 Fernando PU, Viby-Mogensen J, Bonsu AK, *et al*. Relationship between posttetanic count and response to carinal stimulation during vecuronium-induced neuromuscular blockade. *Acta Anaesthesiol Scand* 1987;31: 593-6.

32 Dhonneur G, Kirov K, Motamed C, *et al.* Post-tetanic count at adductor pollicis is a better indicator of early diaphragmatic recovery than train-of-four count at corrugator supercilii. *Br J Anaesth* 2007;99: 376–9.

33 Casanova J, Piñeiro P, De La Gala F, *et al*. Deep versus moderate neuromuscular block during one-lung ventilation in lung resection surgery. *Rev Bras Anestesiol* 2017;67: 288-93.

34 Fortier LP, McKeen D, Turner K, *et al.* The RECITE study: a Canadian Prospective, Multicenter Study of the incidence and severity of residual neuromuscular blockade. *Anesth Analg* 2015; 121:366-72.

35 Raval AD, Uyei J, Karabis A, *et al.* Incidence of residual neuromuscular blockade and use of neuromuscular blocking agents with or without antagonists: A systematic review and meta-analysis of randomized controlled trials. *J Clin Anesth* 2020; 64:109818.

36 Kheterpal S, Vaughn MT, Dubovoy TZ, *et al*. Sugammadex versus Neostigmine for Reversal of Neuromuscular Blockade and Postoperative Pulmonary Complications (STRONGER): A Multicenter Matched Cohort Analysis. *Anesthesiology* 2020; 132:1371-81.

For peer review only - http://bmjopen.bmj.com/site/about/guidelines.xhtml

37 Cammu G. Residual Neuromuscular Blockade and Postoperative Pulmonary Complications: What Does the Recent Evidence Demonstrate? *Curr Anesthesiol Rep* 2020; 27:1-6.

38 Grabitz SD, Rajaratnam N, Chhagani K, *et al*. The Effects of Postoperative Residual Neuromuscular Blockade on Hospital Costs and Intensive Care Unit Admission: A Population-Based Cohort Study. *Anesth Analg* 2019; 128:1129-36.

39 Stawicki N, Gessner P. Residual Neuromuscular Blockade in the Critical Care Setting. *AACN Adv Crit Care*2018;29: 15-24.

40 Saager L, Maiese EM, Bash LD, *et al*. Incidence, risk factors, and consequences of residual neuromuscular block in the United States: The prospective, observational, multicenter RECITE-US study. *J Clin Anesth* 2019; 55:33-41.

41 Shamseer L, Moher D, Clarke M. Preferred reporting items for systematic review and meta-analysis protocols (PRISMA-P) 2015: elaboration and explanation. *BMJ* 2015; 350: g7647.

42 Higgins JPT, Thomas J, Chandler J, *et al*. Cochrane Handbook for Systematic Reviews of Interventions version 6.2 (updated February 2021). Cochrane, 2021. Available: www.training.cochrane.org/handbook.

43 Page MJ, McKenzie JE, Bossuyt PM, *et al*. The PRISMA 2020 statement: an updated guideline for reporting systematic reviews. *BMJ* 2021;372: n71.

44 Brull SJ, Kopman AF. Current Status of Neuromuscular Reversal and Monitoring: Challenges and Opportunities. Anesthesiology. 2017 Jan;126(1):173-190.

45 Martini CH, Boon M, Bevers RF, *et al*. Evaluation of surgical conditions during laparoscopic surgery in patients with moderate vs. deep neuromuscular block. *Br J Anaesth* 2014;112: 498-505.

46 Gheibi S, Mahmoodzadeh A, Kashfi K, *et al*. Data Extraction from Graphs Using Adobe Photoshop: Applications for Meta-Analyses. *Int J Endocrinol Metab* 2019;17: e95216.

47 Higgins JPT, Altman DG, Gøtzsche PC, *et al*. The Cochrane collaboration's tool for assessing risk of bias in randomised trials. *BMJ* 2011; 343: d5928.

48 Higgins JPT, Savović J, Page MJ, et al. Chapter 8: Assessing risk of bias in a

For peer review only - http://bmjopen.bmj.com/site/about/guidelines.xhtml

randomized trial. In: Higgins JPT, Thomas J, Chandler J, Cumpston M, Li T, Page MJ, Welch VA (editors). Cochrane Handbook for Systematic Reviews of Interventions version 6.2 (updated February 2021). Cochrane, 2021. Available from www.training.cochrane.org/handbook.

49 Savović J, Turner RM, Mawdsley D, *et al*. Association between Risk of-Bias assessments and results of randomized trials in Cochrane reviews: the ROBES Meta-Epidemiologic study. *Am J Epidemiol* 2018;187: 1113-22.

50 Wetterslev J, Thorlund K, Brok J, *et al*. Estimating required information size by quantifying diversity in random-effects model meta-analyses. *BMC Med Res Methodol* 2009; 9:86.

51 Wetterslev J, Jakobsen JC, Gluud C. Trial sequential analysis in systematic reviews with meta-analysis. *BMC Med Res Methodol* 2017; 17:39.

52 Wetterslev J, Thorlund K, Brok J, *et al*. Trial sequential analysis may establish when firm evidence is reached in cumulative meta-analysis. *J Clin Epidemiol* 2008; 61: 64-75.

53 Imberger G, Thorlund K, Gluud C, *et al*. False-Positive findings in Cochrane meta-analyses with and without application of trial sequential analysis: an empirical review. *BMJ Open* 2016;6: e011890.

54 Brok J, Thorlund K, Gluud C, *et al*. Trial sequential analysis reveals insufficient information size and potentially false positive results in many meta-analyses. *J Clin Epidemiol* 2008; 61:763-9.

55 Norman GR, Sloan JA, Wyrwich KW. Interpretation of changes in health-related quality of life: the remarkable universality of half a standard deviation. *Med Care* 2003; 41:582-92.

56 Castellini G, Nielsen EE, Gluud C. Comment on: "Cell therapy for heart disease: Trial sequential analyses of two cochrane reviews". *Clin Pharmacol Ther* 2017; 102:21-4.

57 Thorlund K, Engstrøm J, Wetterslev J, *et al.* User manual for trial sequential analysis (TSA) Copenhagen trial unit, centre for clinical intervention research, Denmark; 2011.Available: http://www.ctu.dk/tsa [Accessed 2021].

58 Wetterslev J, Thorlund K, Brok J, *et al.* Estimating required information size by

For peer review only - http://bmjopen.bmj.com/site/about/guidelines.xhtml

quantifying diversity in random-effects model meta-analyses. *BMC Med Res Methodol* 2009; 9:86.

59 Egger M, Davey Smith G, Schneider M, *et al*. Bias in meta-analysis detected by a simple, graphical test. *BMJ* 1997;315: 629-34.

60 Sterne JA, Gavaghan D, Egger M. Publication and related bias in meta-analysis: power of statistical tests and prevalence in the literature. *J Clin Epidemiol* 2000; 53:1119-29.

61 Mavridis D, Salanti G. How to assess publication bias: funnel plot, trim-and-fill method and selection models. *Evid Based Ment Health* 2014; 17:30.

62 Guyatt G, Oxman AD, Akl EA, *et al.* Grade guidelines: 1. Introduction-GRADE evidence profles and summary of fndings tables. *J Clin Epidemiol* 2011; 64:383-94.

Figure Legends

Figure 1. The PRISMA flow diagram.

For peer review only

For peer review only - http://bmjopen.bmj.com/site/about/guidelines.xhtml

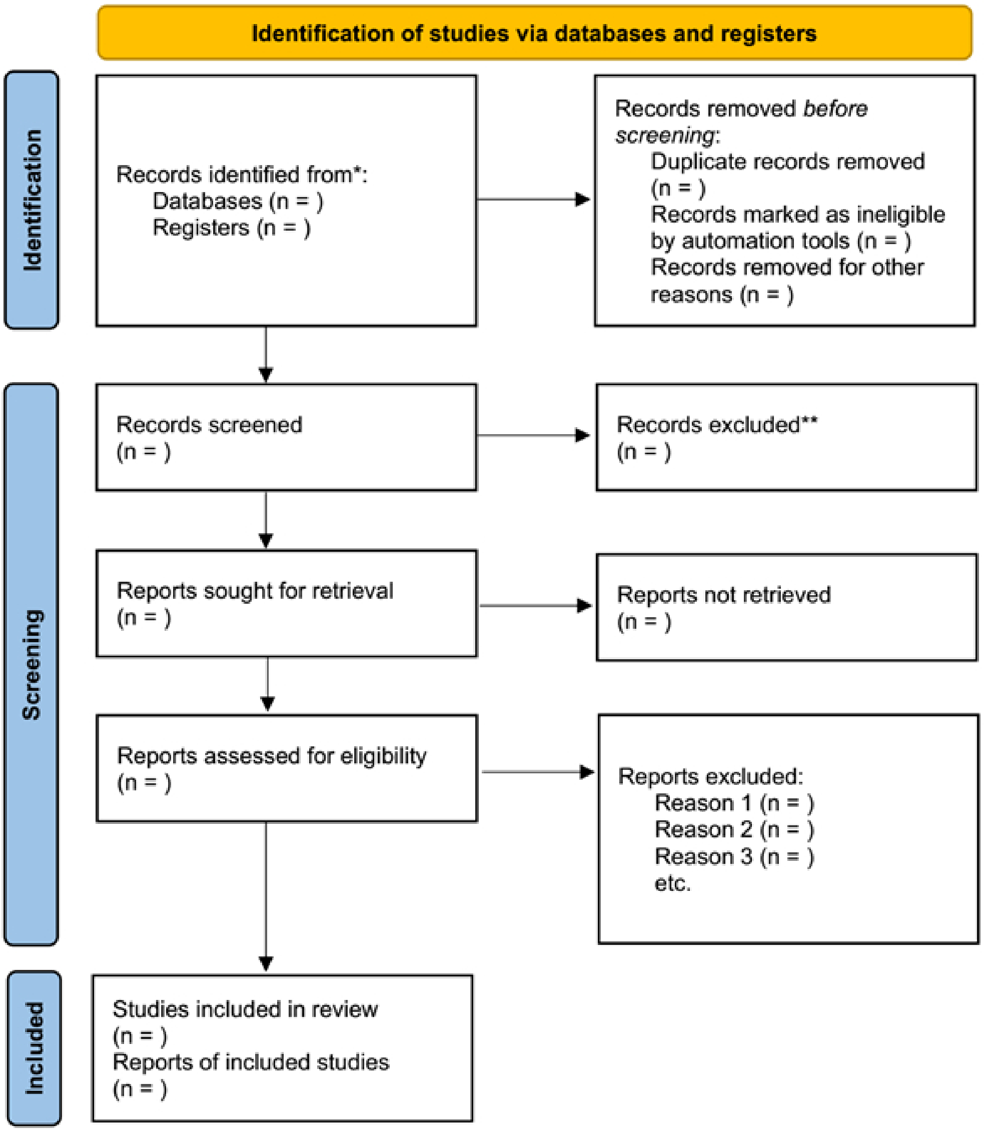

Figure 1 PRISMA_2020_flow_diagram

Supplementary appendix 1: Search strategy

Search strategy of PubMed as follows:

#1 “neuromuscular blockade"[MeSH Terms] OR neuromusc*[tiab]” OR “muscle relaxation” [MeSH Terms]

#2 “Deep[tiab] OR profound[tiab] OR intense[tiab] OR extreme[tiab] OR depth[tiab]”

#3 “Pulmonary” [Mesh] OR “Lung” [Mesh] OR Pulmonary [tiab] OR Lung [tiab]

#4 “Surgical Procedures Operative” [Mesh] OR “Microsurgery” [Mesh] OR “Surgical Procedures Minimally Invasive” [Mesh] OR Minimally Invasive Surgery[tiab] OR MIS [tiab] OR Minimal Access Surgical Procedures [tiab]OR Minimal Surgical Procedures[tiab] OR Minimally Invasive Surgical Procedures [tiab] OR Minimal Surgical Procedure[tiab] OR minimally invasive surgical procedure [tiab] OR minimal access surgical procedure[tiab]

#5 “Thoracic surgery, Video-Assisted” [Mesh] or Surgeries, Video-Assisted Thoracic [af] or Surgery, Video-Assisted Thoracic [af] or Thoracic Surgeries, Video-Assisted [af] or Thoracic surgery, Video-Assisted [af] or Video-Assisted Thoracic Surgeries [af] or Video-Assisted Thoracic Surgery [af] or Surgeries, Video-Assisted Thoracoscopic [af] or Surgery, Video-Assisted Thoracoscopic [af] or Thoracoscopic Surgeries, Video-Assisted [af] or Thoracoscopic Surgery, Video-Assisted [af] or Video Assisted Thoracoscopic Surgery [af] or Video Assisted Thoracoscopic Surgeries [af] or Video-Assisted Thoracic Surgery [af] or Video Assisted Thoracic Surgery [af] or Surgery, Thoracic, Video-Assisted [af] or VATS [af] or VATSs [af].

#6 “Robotics” [MeSH] OR robot* [tiab] OR computer guid*[tiab] OR computer-guid*[tiab] OR computer-assisted[tiab] OR computer assisted [tiab]OR da Vinci [tiab]OR Zeus [tiab]OR telesurgery[tiab]

#7 #1 AND #2 AND #3

#8 #4 OR #5 OR #6

#9 “controlled clinical trial” [Publication Type] OR “randomized controlled trial” [Publication Type] OR “randomized” [Title/Abstract] OR “randomized” [Title/Abstract] OR “Placebo” [Title/Abstract] OR “randomly” [Title/Abstract] OR “Clinical trial” [Title]

#10 (animals [MeSH Terms]) NOT ((human [MeSH Terms]) AND (animals [MeSH Terms]))

#11 #7and #8 and #9 not #10

Search strategy of Cochrane library as follows:

For peer review only

#1 MeSH descriptor: [neuromuscular blockade] explode all trees

#2 MeSH descriptor: [muscle relaxation] explode all trees

#3 (neuromusc*): ti,ab,kw

#4 #1 or # 2 or # 3

#5 (Deep): ti,ab,kw or (profound):ti,ab,kw or (intense):ti,ab,kw or (extreme):ti, ab,kw or (depth):ti,ab,kw

#6 #4 and #5

#7 MeSH descriptor: [Pulmonary] explode all trees

#8 MeSH descriptor: [Lung] explode all trees

#9 #7 or # 8

#10 #6 and #9

#11 MeSH descriptor: [Surgical Procedures Operative] explode all trees

#12 MeSH descriptor: [Thoracic surgery, Video-Assisted] explode all trees

#13 MeSH descriptor: [Microsurgery] explode all trees

#14 MeSH descriptor: [Surgical Procedures Minimally Invasive] explode all trees

#15 MeSH descriptor: [Robotics] explode all trees

#16 (surgery or surgical* or Video-Assisted* or Video Assisted* or Video* or Thorac* or VATS): ti,ab,kw

#17 (robot*): ti,ab,kw

#18 (computer guid* OR computer-guid* OR computer-assisted OR computer assisted): ti,ab,kw

#19 (da Vinci OR Zeus OR telesurgery): ti,ab,kw

#20 #11 or # 12 or #13 or # 14 or #15 or # 16 or #17 or # 18 or #19

#21 #10 and # 20

#22 (controlled clinical trial):pt or (randomized controlled trial):pt or (random*): ti,ab,kw or (Clinical trial):ti,ab,kw

#23 #21 and #22

Search strategy of Web of Science as follows:

#1 TS= (neuromuscular blockade or neuromusc* or muscle relaxation)

#2 TS= (Deep or profound or intense or extreme or depth)

#3 TS= (Pulmonary or Lung)

#4 TS= (Surgical Procedures Operative or Thoracic surgery, Video-Assisted or Microsurgery or Surgical Procedures Minimally Invasive or Robotics)

#5TS= (surgery or surgical* or Video-Assisted* or Video Assisted* or Video* or Thorac* or VATS or robot* or computer guid* or computer-guid* or computer-assisted or computer assisted or da Vinci or Zeus or telesurgery)

#6 #4 OR #5

#7 #1 and #2 and #3 and #6

#8 TS= (random* or Clinical trial)

#9 #7 and #8

Search strategy for Ovid Medline as follows:

#1 exp neuromuscular blockade/ or exp muscle relaxation/ or neuromusc*.mp.

#2 (Deep or profound or intense or extreme or depth) .mp.

#3 exp pulmonary / or exp Lung

#4 #1 and #2 and #3

#5 exp surgical procedures operative/ or exp Thoracic surgery, Video-Assisted / or exp Microsurgery/or exp Surgical Procedures Minimally Invasive/ or exp Robotics/

#6 (surgery or surgical*).mp.

#7 (Video-Assisted* or Video Assisted* or Video* or Thorac* or VATS).mp.

#8 (robot*).mp.

#9(computer guid* or computer-guid* or computer-assisted or computer assisted).mp.

#10(da Vinci or Zeus or telesurgery).mp.

#11 #5or #6 or #7 or #8 or #9 or #10

#12 randomized controlled trial.pt.

#13 controlled clinical trial.pt.

#14 randomized.ab.

#15 placebo.ab.

#16 clinical trials as topic.sh.

#17 randomly.ab.

#18 trial.ti.

#19 #12 or #13 or #14 or #15 or #16 or #17 or #18

For peer review only

#20 (animals not (humans and animals)).sh.

#21 #19 not #20

#22 #4 and #11 and #21

Search strategy for Embase as follows:

#1 exp neuromuscular blockade/

#2 neuromusc*.mp.

#3 exp muscle relaxation/

#4 (Deep or profound or intense or extreme or depth).mp.

#5 exp Pulmonary /

#6 exp Lung/

#7 exp Surgical Procedures Operative/

#8 exp Thoracic surgery, Video-Assisted /

#9 exp Microsurgery/

#10 exp Surgical Procedures Minimally Invasive/

#11 exp Robotic/

#12 (surgery or surgical*).mp.

#13 (robot*).mp.

#14(Video-Assisted* or Video Assisted* or Video* or Thorac* or VATS).mp.

#15 (computer guid* or computer-guid* or computer-assisted or computer assisted).mp.

#16 (da Vinci or Zeus or telesurgery).mp.

#17 Clinical trial.mp.

#18 (placebo*).mp.

#19 exp randomized controlled trial/

#20 (random*).mp.

#21 (exp animal/ or nonhuman/ or exp animal experiment/) not human/

#22 #1 or #2 or #3

#23 #22 and #4

#24 #5 or #6

#25 #7 or #8 or #9 or #10 or #11 or #12 or #13 or #14 or #15 or #16

#26 #23 and #24 and #25

For peer review only

#27 #17 or #18 or #19 or #20

#28 #26 and #27

#29 #28 not #21

WHO ICTRP Trial registry

http://apps.who.int/trialsearch (WHO ICTRP register) will be searched via the advanced search page. Search terms were: (Lung or Pulmonary) AND (minimal invasive or minimally invasive or surgery or surgical procedures operative or microsurgery or Video-Assisted* or Video Assisted* or Video* or Thorac* or VATS or robot* or computer guid* or computer-guid* or computer-assisted or computer assisted or da Vinci or Zeus or telesurgery) AND (Deep neuromuscular blockade or profound neuromuscular blockade or intense neuromuscular blockade or extreme neuromuscular blockade or depth neuromuscular blockade).

Clinicaltrials.gov search strategy

http://clinicaltrials.gov (NIH register) will be searched via advanced search page. Search terms were: Condition or disease: (Lung or Pulmonary) AND (minimal invasive or minimally invasive or surgery or surgical procedures operative or microsurgery or Video-Assisted* or Video Assisted* or Video* or Thorac* or VATS or robot* or computer guid* or computer-guid* or computer-assisted or computer assisted or da Vinci or Zeus or telesurgery).

Study type: Interventional Studies.

Intervention/treatment: (Deep neuromuscular blockade or profound neuromuscular blockade or intense neuromuscular blockade or extreme neuromuscular blockade or depth neuromuscular blockade)

Chinese database

China National Knowledge Infrastructure (CNKI) search strategy

(电视胸腔镜 or 胸腔镜 or 腔镜 or 微创 or 机器人 or 机器人辅助 or 达芬奇 or 宙斯 or RATS or VATS or 多孔 VATS or 四孔 VATS or 三孔 VATS or 两孔 VATS or 单孔 VATS) and (手术 or 切除术 or 根治术) and (肺 or 肺癌 or 肺肿瘤) and (深度肌松 or 深肌松 or 深度神经肌肉阻滞)and (随机 or 对照)

Chinese BioMedical Literature (CBM)

("电视胸腔镜" [全部字段] or"胸腔镜" [全部字段] or "腔镜" [全部字段] or "微创" [全部字段]

For peer review only

or "机器人" [全部字段] or "机器人辅助" [全部字段] or "达芬奇" [全部字段] or "宙斯" [全部字段] or " RATS " [全部字段] or "VATS [全部字段] or "多孔 VATS" [全部字段]or "四孔 VATS " [全部字段] or "三孔 VATS" [全部字段] or "两孔 VATS" [全部字段] or "单孔 VATS" [全部字段]) and ("手术" [全部字段] or "切除术" [全部字段] or"根治术" [全部字段]) and ("肺" [全部字段] or "肺癌" [全部字段] or"肺肿瘤" [全部字段]) and ("深度肌松" [全部字段] or "深肌松" [全部字段] or"深度神经肌肉阻滞" [全部字段]) and ("随机" [全部字段] or "对照" [全部字段])

VIP database

关键词=(电视胸腔镜 or 胸腔镜 or 腔镜 or 微创 or 机器人 or 机器人辅助 or 达芬奇 or 宙斯 or RATS or VATS or 多孔 VATS or 四孔 VATS or 三孔 VATS or 两孔 VATS or 单孔 VATS) AND 关键词= (手术 or 切除术 or 根治术) AND 关键词= (肺 or 肺癌 or 肺肿瘤) AND 关键词= (深度肌松 or 深肌松 or 深度神经肌肉阻滞)AND 关键词= (随机 or 对照)

Wan fang database.

(电视胸腔镜 or 胸腔镜 or 腔镜 or 微创 or 机器人 or 机器人辅助 or 达芬奇 or 宙斯 or RATS or VATS or 多孔 VATS or 四孔 VATS or 三孔 VATS or 两孔 VATS or 单孔 VATS) and (手术 or 切除术 or 根治术) and (肺 or 肺癌 or 肺肿瘤) and (深度肌松 or 深肌松 or 深度神经肌肉阻滞)and (随机 or 对照)

For peer review only

Assessment of risk of bias in included studies

Random sequence generation

- ***Low risk:*** If sequence generation was achieved using computer random number generator or a random number table. Drawing lots, tossing a coin, shuffling cards, and throwing dice were also considered adequate if performed by an independent adjudicator.
- ***Unclear risk:*** If the method of randomisation was not specified, but the trial was still presented as being randomised.
- ***High risk:*** If the allocation sequence is not randomised or only quasi-randomised. These trials will be excluded.

Allocation concealment

- ***Low risk:*** If the allocation of patients was performed by a central independent unit, onsite locked computer or identical-looking numbered sealed envelopes.
- ***Uncertain risk:*** If the trial was classified as randomised but the allocation concealment process was not described.
- ***High risk:*** If the allocation sequence was familiar to the investigators who assigned participants.

Blinding of participants and treatment providers

- ***Low risk:*** If the participants and the treatment providers were blinded to intervention allocation and this was described.
- ***Uncertain risk:*** If the procedure of blinding was insufficiently described.
- ***High risk:*** If blinding of participants and the treatment providers was not performed.

Blinding of outcome assessment

- ***Low risk of bias:*** If it was mentioned that outcome assessors were blinded and this was described.
- ***Uncertain risk of bias:*** If it was not mentioned if the outcome assessors in the trial were blinded or the extent of blinding was insufficiently described.
- ***High risk of bias:*** If no blinding or incomplete blinding of outcome assessors was performed.

Incomplete outcome data

- ***Low risk of bias:*** If missing data were unlikely to make treatment effects depart from plausible

For peer review only

values. This could be either (1) there were no drop-outs or withdrawals for all outcomes, or (2) the numbers and reasons for the withdrawals and drop-outs for all outcomes were clearly stated and could be described as being similar to both groups. Generally, the trial is judged as at a low risk of bias due to incomplete outcome data if drop-outs are less than 5%. However, the 5% cut-off is not definitive.

- ***Uncertain risk of bias:*** If there was insufficient information to assess whether missing data were likely to induce bias on the results.
- ***High risk of bias:*** If the results were likely to be biased due to missing data either because the pattern of drop-outs could be described as being different in the two intervention groups or the trial used improper methods in dealing with the missing data (e.g. last observation carried forward).

Selective outcome reporting

- ***Low risk of bias:*** If a protocol was published before or at the time the trial was begun and the outcomes specified in the protocol were reported on. If there is no protocol or the protocol was published after the trial has begun, reporting of serious adverse events will grant the trial a grade of low risk of bias.
- ***Uncertain risk of bias:*** If no protocol was published and the outcome of serious adverse events were not reported on.
- ***High risk of bias:*** If the outcomes in the protocol were not reported on.

Other risks of bias

- ***Low risk of bias:*** If the trial appears to be free of other components (for example, academic bias or for-profit bias) that could put it at risk of bias.
- ***Unclear risk of bias:*** If the trial may or may not be free of other components that could put it at risk of bias.
- ***High risk of bias:*** If there are other factors in the trial that could put it at risk of bias (for example, authors conducted trials on the same topic, for- profit bias, etc.).

Overall risk of bias

- ***Low risk of bias:*** The trial will be classified as overall 'low risk of bias' only if all of the bias domains described in the above paragraphs are classified as 'low risk of bias'.
- ***High risk of bias:*** The trial will be classified as 'high risk of bias' if any of the bias risk domains

For peer review only

described in the above are classified as 'unclear' or 'high risk of bias'.

- We will assess the domains 'blinding of outcome assessment', 'incomplete outcome data', and 'selective out- come reporting' for each outcome result. Thus, we can assess the bias risk for each outcome assessed in addition to each trial. Our primary conclusions will be based on the results of our primary outcome results with overall low risk of bias. Both our primary and secondary conclusions will be presented in the summary of findings tables.

For peer review only

PRISMA-P checklist

Table PRISMA-P (preferred reporting items for systematic review and meta-analysis protocols) 2015 checklist: recommended items to address in a systematic review protocol

Section and topic	Item No	Checklist item	Reported on page #
**Administrative information**			
Title:			
Identification	1a	Identify the report as a protocol of a systematic review	1
Update	1b	If the protocol is for an update of a previous systematic review, identify as such	None
Registration	2	If registered, provide the name of the registry (such as PROSPERO) and registration number	3,6
Authors:			
Contact	3a	Provide name, institutional affiliation, e-mail address of all protocol authors; provide physical mailing address of corresponding author	1
Contributions	3b	Describe contributions of protocol authors and identify the guarantor of the review	19
Amendments	4	If the protocol represents an amendment of a previously completed or published protocol, identify as such and list changes; otherwise, state plan for documenting important protocol amendments	None
Support:			
Sponsor	5b	Provide name for the review funder and/or sponsor	None
Role of sponsor or funder	5c	Describe roles of funder(s), sponsor(s), and/or institution(s), if any, in developing the protocol	None
**Introduction**			
Rationale	6	Describe the rationale for the review in the context of what is already known	3-6
Objectives	7	Provide an explicit statement of the question(s) the review will address with reference to participants, interventions, comparators, and outcomes (PICO)	6-8
**Methods**			
Eligibility criteria	8	Specify the study characteristics (such as PICO, study design, setting, time frame) and report characteristics (such as years considered, language, publication status) to be used as criteria for eligibility for the review	6-9

Information sources	9	Describe all intended information sources (such as electronic databases, contact with study authors, trial registers or other grey literature sources) with planned dates of coverage	9-11
Search strategy	10	Present draft of search strategy to be used for at least one electronic database, including planned limits, such that it could be repeated	10-11, S1
Study records:			
Data management	11a	Describe the mechanism(s) that will be used to manage records and data throughout the review	11
Selection process	11b	State the process that will be used for selecting studies (such as two independent reviewers) through each phase of the review (that is, screening, eligibility and inclusion in meta-analysis)	12-13
Data collection process	11c	Describe planned method of extracting data from reports (such as piloting forms, done independently, in duplicate), any processes for obtaining and confirming data from investigators	12-13
Data items	12	List and define all variables for which data will be sought (such as PICO items, funding sources), any pre-planned data assumptions and simplifications	11-12
Outcomes and prioritization	13	List and define all outcomes for which data will be sought, including prioritization of main and additional outcomes, with rationale	8-9
Risk of bias in individual studies	14	Describe anticipated methods for assessing risk of bias of individual studies, including whether this will be done at the outcome or study level, or both; state how this information will be used in data synthesis	13-16
Data synthesis	15a	Describe criteria under which study data will be quantitatively synthesised	15-16
	15b	If data are appropriate for quantitative synthesis, describe planned summary measures, methods of handling data and methods of combining data from studies, including any planned exploration of consistency (such as I^2, Kendall's τ)	16
	15c	Describe any proposed additional analyses (such as sensitivity or subgroup analyses, meta-regression)	15-17
	15d	If quantitative synthesis is not appropriate, describe the type of summary planned	14-15
Meta-bias(es)	16	Specify any planned assessment of meta-bias(es) (such as publication bias across studies, selective reporting within studies)	18
Confidence in cumulative evidence	17	Describe how the strength of the body of evidence will be assessed (such as GRADE)	14-17

BMJ Open

Deep neuromuscular block for minimally invasive lung surgery: a protocol for a systematic review with meta-analysis and trial sequential analysis

Journal:	*BMJ Open*
Manuscript ID	bmjopen-2021-056816.R2
Article Type:	Protocol
Date Submitted by the Author:	11-Apr-2022
Complete List of Authors:	Zheng, Jianqiao; Sichuan University West China Hospital, Department of Anesthesiology<br>Du, Li; University of Electronic Science and Technology of China, Department of Anesthesiology<br>Deng, Xiaoqian; Sichuan University West China Hospital, Department of Anesthesiology<br>Zhang, Lu; Sichuan University West China Hospital, Department of Anesthesiology<br>Wang, Jia; Sichuan University West China Hospital, Department of Anesthesiology<br>Chen, Guo; Sichuan University West China Hospital, Department of Anesthesiology
**Primary Subject Heading**:	Surgery
Secondary Subject Heading:	Anaesthesia, Surgery
Keywords:	Cardiothoracic surgery < SURGERY, Adult anaesthesia < ANAESTHETICS, Adult surgery < SURGERY

SCHOLARONE™
Manuscripts

TITLE PAGE

Deep neuromuscular block for minimally invasive lung surgery: a protocol for a systematic review with meta-analysis and trial sequential analysis

Authors

Jianqiao Zheng[1] E-mail: zhjq1983@163.com

Li Du[2] E-mail: huaying-duli@163.com

Xiaoqian Deng[1] E-mail: 50058837@qq.com

Lu Zhang[1] E-mail: 304022514@qq.com

Jia Wang[1] E-mail: 19983157894@qq.com

*Guo Chen[1] E-mail: Anesthesiology_SCU@163.com

[1]Department of Anesthesiology, West China Hospital, Sichuan University No. 37th, Guoxue Alley, Wuhou District, Chengdu 610041, Sichuan, China.

[2]Department of Anesthesiology, Sichuan Cancer Hospital & Institute, Sichuan Cancer Center, School of Medicine, University of Electronic Science and Technology of China, No.55th, People's South Road, Chengdu 610041, Sichuan, China.

*Corresponding Author

Name: Guo Chen

E-mail: Anesthesiology_SCU@163.com

Phone: +86-28-8542-3593

Address: Department of Anesthesiology, Sichuan University West China Hospital, No. 37th, Guoxue Alley, Wuhou District, Chengdu 610041, Sichuan, China.

Words: 3628

For peer review only

For peer review only - http://bmjopen.bmj.com/site/about/guidelines.xhtml

ABSTRACT

Introduction Minimally invasive lung surgery (MILS) gradually became the primary surgical therapy for lung cancer, which remains the leading cause of cancer death. Adequate muscle relaxation by deep neuromuscular block (NMB) is particularly necessary for MILS to provide a satisfactory surgical field. However, deep NMB for MILS remains controversial, as one-lung ventilation may provide an acceptable surgical field. Then, we will perform a protocol for a systematic review and meta-analysis to identify the efficacy of deep NMB for MILS.

Methods and analysis We will search the PubMed, Cochrane Library, Embase, Ovid Medline, Web of Science, Chinese BioMedical Literature, China National Knowledge Infrastructure, VIP and Wanfang databases from inception to March 2022 to identify randomized controlled trials of adult participants undergoing MILS with deep NMB. Studies published in English or Chinese will be considered. The primary outcome will be the surgical conditions according to the surgeon's perspective. Secondary outcomes will be the incidence of perioperative events and perioperative mortality. Heterogeneity will be assessed by the χ^2 test and I^2 statistic. Data will be synthesized by both a fixed-effect and a random-effects meta-analysis, with an intention to present the random-effects result if there is no indication of funnel plot asymmetry. Otherwise, meta-regression will be used. The Cochrane risk-of-bias tool, trial sequential analysis and

For peer review only - http://bmjopen.bmj.com/site/about/guidelines.xhtml

GRADE (Grading of Recommendations Assessment, Development and Evaluation) will be used to assess the evidence quality and control the risks of random errors. Funnel plots and Egger's regression test will be used to assess publication bias.

Ethics and dissemination Ethical approval was not required for this systematic review protocol. The results will be disseminated through peer-reviewed publications.

Keywords deep neuromuscular block, minimally invasive, thoracoscopic, pulmonary, meta-analysis, randomized controlled trial.

PROSPERO registration number CRD42021254016

Strengths and limitations of the study

► This systematic review protocol according to the Preferred Reporting Items for Systematic Review and Meta-Analysis Protocols (PRISMA-P) guidelines to perform a rigorous risk of bias assessment.

► Trial sequential analysis will be performed to control the risks of false positives by estimating the diversity adjusted information size for the outcomes.

► Funnel plots and Egger's regression test will be applied to assess publication bias.

► Heterogeneity will be assessed by subgroup analysis based on participants' age, body mass index, and type of MILS.

For peer review only - http://bmjopen.bmj.com/site/about/guidelines.xhtml

INTRODUCTION

Lung cancer remains the leading cause of cancer death, with an estimated 1.8 million new deaths in 2020, accounting for 18% of the total cancer deaths according to Global Cancer Statistics 2020. [1] Lung cancer is the second most common cancer, with an estimated 2.2 million new cases in 2020, representing 11.4% of all cancer cases. [1] Due to its the high incidence and mortality, the treatment of lung cancer is a global challenge.

Surgical resection remains the primary therapy in the treatment of lung cancer. Since the 1990s, minimally invasive surgical techniques of video-assisted thoracic surgery and robotic-assisted thoracic surgery have been applied in the diagnosis and treatment of intrathoracic diseases. [2-4] Growing experience with minimally invasive lung surgery (MILS), combined with improvements in video technology and instrumentation, has allowed conventional thoracotomy to be gradually replaced by MILS in recent years.[5-8]

Recent literature suggests that MILS was equivalent to open thoracotomy on long-term survival and overall oncologic efficacy, even with a better short-term survival.[9-15] The minimally invasive surgical approach is still the favored surgical procedure in that it offers many advantages, including less trauma and pain, faster recovery, fewer complications, lower immunological responses, and a shorter hospitalization period. [16-21] In addition, it is associated with a higher

For peer review only - http://bmjopen.bmj.com/site/about/guidelines.xhtml

tolerance to postoperative adjuvant therapy and mitigates or ameliorates the postoperative decline in health-related functional status. [22-25]

Adequate muscle relaxation by deep neuromuscular block (NMB) is particularly necessary for minimally invasive surgical techniques.[26-28] MILS involves areas adjacent to major blood vessels and can trigger intraoperative body movement, cough, and diaphragm movement.[29] Moreover, the diaphragm is the most resistant muscle to neuromuscular blocking agents (NMBAs), and movement of the diaphragm can interfere with the surgical procedure. Deep NMB can inhibit the response to carinal stimulation and prevent bucking and coughing during surgical procedures. [30-32] In addition, it can reduce the peak pressure and plateau pressure and improve lung compliance and peripheral oxygen saturation during one-lung ventilation. [33]

There is still controversy regarding the clinical benefit of maintaining deep NMB for MILS because deep NMB seems unnecessary, as ribcage provides thoracic support and one-lung ventilation usually provides a satisfactory surgical field. In addition, the risk of residual neuromuscular block is estimated to occur in 26% to 88% of patients undergoing general anesthesia, and this incidence is inevitably increased after deep NMB. [34 35] Numerous clinical studies have documented that postoperative residual neuromuscular block has the potential risk of increasing the incidence of postoperative pulmonary complications (such as airway obstruction,

For peer review only - http://bmjopen.bmj.com/site/about/guidelines.xhtml

aspiration, and hypoxia), the odds of hospital readmission intensive care unit admission and the hospital length of stay. [36-40]

Hence, the clinical benefits of deep NMB for MILS remain controversial. Therefore, it is necessary to conduct a systematic review and meta-analysis to analyze the clinical efficacy of deep NMB on MILS. The outcomes of this systematic review will provide evidence for better clinical decision making and possible directions for further clinical trials.

Objectives

We are performing this protocol of systematic review and meta-analysis to determine the clinical efficacy of deep NMB on the surgical conditions of MILS according to the surgeon's perspective. Patients' postoperative recovery and the incidence of perioperative events will also be identified. Furthermore, trial sequential analysis (TSA) will be applied to confirm the reliability of the results.

METHODS AND ANALYSIS

Study design

Our review protocol was registered with PROSPERO 2021 (registration number: CRD 42021254016). This protocol was designed according to the Preferred Reporting Items for Systematic Reviews and Meta-Analyses Protocols (PRISMA-P) guidelines.[41] The systematic review and meta-analysis will be performed according to the Cochrane Handbook and reported in accordance with the PRISMA statement. [42 43]

For peer review only - http://bmjopen.bmj.com/site/about/guidelines.xhtml

The study is anticipated to begin searching in March 2022 and complete in May 2022.

Inclusion/exclusion criteria for study selection

Types of studies

We will include all randomized controlled trials (RCTs) involving the efficacy of deep NMB for MILS. Only studies published in English or Chinese will be included.

Studies will be excluded as follows: (1) studies without a control group, compared deep NMB produced by different kinds of NMBAs only; (2) studies with incorrect data obviously, incomplete data or study data that cannot be used for statistical analysis; and (3) studies that were abstracts from conferences, letters, editorials, reviews, observational studies, retrospective studies, and duplicate publications.

Types of participants

Adult participants (≥18 years old) undergoing any kind of MILS (including thoracoscopic surgery, video-assisted thoracic surgery or robotic-assisted thoracic surgery) with deep NMB will be included. No limitations will be defined on participants' characteristics including gender, ethnicity, and body mass index (BMI).

Types of interventions/controls

The intervention group will be the participants who received deep NMB [defined as a train-of-four (TOF) count of zero and a post-tetanic

For peer review only - http://bmjopen.bmj.com/site/about/guidelines.xhtml

count (PTC) ≥1] and intense (profound) NMB [defined as a train-of-four (TOF) count =0 and a post-tetanic count (PTC) =0] throughout the MILS.[44]

In the control group, participants had to receive shallow NMB (defined as a TOF count =4 or measured TOF ratio= 0.1-0.4), moderate NMB (defined as TOF count=1-3) or without NMBAs throughout the MILS. [44]

Types of outcome measures

We will perform the meta-analysis only if at least 2 RCTs have been published in the literature.

Primary outcomes

The primary outcome will be the surgical conditions of the MILS according to the surgeon's perspective. Surgical conditions were evaluated as a surgical rating scale or the percentage of patients with clinically acceptable surgical conditions (Clinically acceptable surgical conditions were defined as Acceptable, Good or Optimal conditions) (table 1).[45]

Table 1 Surgical rating scale (SRS)

SRS category (scale)	Conditions Description
Extremely poor conditions (Score 1)	The surgeon is unable to work because of coughing or of the inability to obtain a visible field because of inadequate muscle relaxation.
Poor conditions (Score 2)	There is a visible field, but the surgeon is severely hampered by inadequate muscle relaxation with continuous muscle contractions, movements, or both.
Acceptable conditions (Score 3)	There is a wide visible field but muscle contractions, movements, or both occur regularly
Good conditions (Score 4)	A wide working field with sporadic muscle contractions, movements, or both
Optimal conditions (Score 5)	A wide visible working field without any movement or contractions.

Secondary outcomes

For peer review only - http://bmjopen.bmj.com/site/about/guidelines.xhtml

1. The incidence of perioperative events included the following:

►Incidence of intraoperative events: defined as body movement, coughing, and breathing against the ventilator (with the aid of airway pressure monitoring and capnography).

►Incidence of postoperative pulmonary complications: defined as the composite of any respiratory infection, respiratory failure, pleural effusion, atelectasis, or pneumothorax.

2. Perioperative mortality

►defined as all-cause death during the operation procedure, within 30 days after surgery, or death during hospitalization.

3. Patients' postoperative recovery

►Recovery time of NMB: defined as the time from administration of the reversal agent to the achievement of a TOF ratio of 0.9.

►Incidence of residual neuromuscular block (defined as TOF <0.90 after tracheal extubation/arrival at PACU.

4. Duration of surgery

Search strategy

We will search English and Chinese electronic databases from inception to March 2022 for published literature. The English databases included PubMed, Cochrane Library, Embase, Ovid Medline and Web of Science. The Chinese databases included the China National Knowledge Infrastructure (CNKI), Chinese BioMedical Literature (CBM), Wanfang

For peer review only - http://bmjopen.bmj.com/site/about/guidelines.xhtml

database and VIP Database. We will also scrutinize the reference lists of each study and trial registry database (Clinical Trials.gov and WHO International Clinical Trials Registry Platform) for missing studies and ongoing or unpublished clinical trials. After data extraction, we will ask the corresponding authors of each included literature for more original data to prevent potential missing data as far as possible.

An example of the search strategy used in PubMed is shown in table 2. The search terms will be used as follows: deep neuromuscular block, minimally invasive, thoracoscopic, video assisted, robotic assisted, pulmonary, and randomized controlled trial. We will translate the search terms into Chinese for literature research and study identification in Chinese databases. Before the final publication of the systematic review, a latest search in the databases will be performed to check if there are any studies published during the preparation of the systematic review. The preliminary search strategy is listed as online supplementary additional file 1.

Table 2 Search strategy for PubMed

No	Search terms
#1	"Neuromuscular blockade"[MeSH] OR neuromusc*[tiab] OR "muscle relaxation" [MeSH]
#2	Deep[tiab] OR profound[tiab] OR intense[tiab] OR extreme[tiab] OR depth[tiab]
#3	"Pulmonary" [Mesh] OR "Lung" [Mesh] OR Pulmonary [tiab] OR Lung [tiab]
#4	"Surgical Procedures Operative" [Mesh] OR "Microsurgery" [Mesh] OR "Surgical Procedures Minimally Invasive" [Mesh] OR Minimally Invasive Surgery[tiab] OR MIS [tiab] OR Minimal Access Surgical Procedures [tiab]OR Minimal Surgical Procedures[tiab] OR Minimally Invasive Surgical

For peer review only - http://bmjopen.bmj.com/site/about/guidelines.xhtml

	Procedures [tiab] OR Minimal Surgical Procedure[tiab] OR minimally invasive surgical procedure [tiab] OR minimal access surgical procedure[tiab]
#5	“Thoracic surgery, Video-Assisted” [Mesh] or Surgeries, Video-Assisted Thoracic [af] or Surgery, Video-Assisted Thoracic [af] or Thoracic Surgeries, Video-Assisted [af] or Thoracic surgery, Video-Assisted [af] or Video-Assisted Thoracic Surgeries [af] or Video-Assisted Thoracic Surgery [af] or Surgeries, Video-Assisted Thoracoscopic [af] or Surgery, Video-Assisted Thoracoscopic [af] or Thoracoscopic Surgeries, Video-Assisted [af] or Thoracoscopic Surgery, Video-Assisted [af] or Video Assisted Thoracoscopic Surgery [af] or Video Assisted Thoracoscopic Surgeries [af] or Video-Assisted Thoracic Surgery [af] or Video Assisted Thoracic Surgery [af] or Surgery, Thoracic, Video-Assisted [af] or VATS [af] or VATSs [af].
#6	“Robotics” [MeSH] OR robot* [tiab] OR computer guid*[tiab] OR computer-guid*[tiab] OR computer-assisted[tiab] OR computer assisted [tiab]OR da Vinci [tiab]OR Zeus [tiab]OR telesurgery[tiab]
#7	#1 AND #2 AND #3
#8	#4 OR #5 OR #6
#9	“Controlled clinical trial” [Publication Type] OR “randomized controlled trial” [Publication Type] OR “randomized” [Title/Abstract] OR “randomized” [Title/Abstract] OR “Placebo” [Title/Abstract] OR “randomly” [Title/Abstract] OR “Clinical trial” [Title]
#10	“animals” [MeSH] NOT (“human” [MeSH] AND “animals” [MeSH])
#11	#7and #8 and #9 not #10

Data collection and analysis

Selection of studies

Two reviewers (Z-JQ and WJ) will be responsible for screening of the retrieved studies independently. Duplicate studies and those not matching the inclusion criteria will be excluded by reading titles and abstracts briefly. Studies meeting the inclusion criteria will be included after reading the full text of each study thoroughly. Any disagreements will be resolved by consulting a third reviewer (DL) as much as possible. A fourth reviewer (CG) will check out all procedures carefully before confirming the data

For peer review only - http://bmjopen.bmj.com/site/about/guidelines.xhtml

extraction. The entire study selection process is detailed in the PRISMA flow diagram (figure 1).

Data extraction

Two reviewers (Z-JQ and ZL) will extract data independently from each included study following a standardized data extraction form (Excel version 2013, Microsoft Inc, Washington DC, USA). Extracted information including participants' demographic data, type of MILS, inclusion and exclusion criteria, level of NMB during MILS (definition and measurement), outcomes (including primary outcomes, secondary outcomes, and exploratory outcomes), etc. Study design (including randomization, allocation concealment, blinding, data collection and statistical analysis, outcome reporting) will also be recorded for the subsequent quality assessment. Continuous resulting data will be recorded as the mean± SD, and dichotomous data will be recorded as the proportion of participants with percentages. If necessary, a third reviewer (D-XQ) will cross-check the data to ensure precision. If information and data were missing or incomplete, we will contact authors of the literature to obtain the original data via email. If necessary, numerical data from graphs will be extracted by Adobe Photoshop as described by Gheibi et al.[46] A detailed extraction list of information and data is presented in table 3.

Table 3 Data and information extraction schedule

**Subject**	**Content**

For peer review only - http://bmjopen.bmj.com/site/about/guidelines.xhtml

Publication information	Name of the first author; contact email; publish year; country; corporate sponsorship.
Participant	Sample size; Age; Sex; Height and weight or body mass index (BMI); American Society of Anesthesiologists (ASA) physical status classification levels; Type of MILS; Inclusion and exclusion criteria if necessary.
Intervention	Level of NMB (deep NMB, intense NMB or profound NMB); Assessment of the NMB level (equipment of neuromuscular function monitor; monitor position); Type of neuromuscular blocking agents (NMBAs); Dose and administration of NMBAs; Administration of NMBAs antagonist (sugammadex or neostigmine).
Control	Level of NMB (moderate NMB; shallow NMB or without NMBAs); Assessment of the DNMB (equipment for neuromuscular function monitor; monitor position); Type of neuromuscular blocking agents (NMBAs); Dose and administration of NMBAs; Administration of NMBAs antagonist (sugammadex or neostigmine).
Outcome	Primary outcome (Surgical rating scale or the percentage of patients with clinically acceptable surgical conditions); Secondary outcome measurements (Perioperative events; Perioperative mortality; Patients' postoperative recovery; Duration of surgery).
Study design	Application of randomization and blinding; Description about allocation concealment; Statistical analysis; Sample size calculation; Outcome reporting.
Other information	Intraoperative temperatures; Bispectral index (BIS) values; Time or condition of tracheal intubation and extubation; Type of anesthesia maintenance technique (Inhalation anesthesia; Total intravenous anesthesia; or both); Duration of anesthesia.

Quality assessment

Two reviewers (WJ and ZL) will assess the risk of bias in each included study under the guidance of the Cochrane risk of bias tool independently.[47] We will evaluate the methodology in domains of random sequence generation, allocation concealment, blinding of participants and personnel, blinding of outcome assessment, incomplete outcome data, selective outcome reporting, other risks of bias and overall risk of bias. The risk of bias components will be divided into three levels (low risk, unclear and high risk) according to the checklist item. If all risk of bias domains were scored as having a low risk of bias, the trial was defined as having a

For peer review only - http://bmjopen.bmj.com/site/about/guidelines.xhtml

low overall risk of bias. If one or more of the bias domains were scored as unclear or high risk of bias, the trial was defined as having a high overall risk of bias. Trials with a low risk of bias in all domains (including sequence generation, allocation concealment, blinding, incomplete data, selective outcome reporting, and other risks of bias) will be classified as having a low overall risk of bias. Trials with one or more of these domains scored as unclear or high risk of bias will be defined as having a high overall risk of bias.[48 49] Disagreements, if any, the risk assignment will be settled through arbitration of a third reviewer (CG). Classification of the trials will follow criteria defined in online supplemental additional file 2.

Measures of treatment effect

Mean differences (MDs) (outcome data reported by same scale) or the standardized mean difference (SMDs) (outcome data reported by different scales) with 95% confidence intervals (CIs) will be used for continuous outcome data. While the relative risks (RRs) with 95% CIs will be used for dichotomous data.

Assessment of heterogeneity

The choice between a fixed-effect and a random-effects meta-analysis based on statistical heterogeneity is not recommended by the Cochrane guidelines.[42] To test the results by the traditional meta-analysis method based on statistical heterogeneity (statistical heterogeneity will be assessed by the standard χ^2 test and I^2 test. If $p \geq 0.1$ and $I^2 \leq 50\%$, the fixed-effects

For peer review only - http://bmjopen.bmj.com/site/about/guidelines.xhtml

model will be used. If $p<0.1$ or $I^2>50\%$, the random-effects model will be used), a pragmatic approach will be performed to undertake both a fixed-effect and a random-effects meta-analysis for each outcome, with the intention of presenting the random-effects result if there is no indication of funnel plot asymmetry. [42] If there is an indication of funnel plot asymmetry, then both methods are problematic. It may be reasonable to present both analyses or neither, or to perform a sensitivity analysis in which small studies are excluded or addressed directly using meta-regression. A P value <0.05 was assumed to be statistically significant.

Trial Sequential Analysis

We will perform trial sequential analysis (TSA), using the TSA program version 0.9.5.10 Beta (Copenhagen Trial Unit, Copenhagen, Denmark) to correct the risks of random errors by calculating the required information size (RIS). [50-52] The RIS is defined as the number of participants required in the meta-analysis to detect or reject the intervention effect. [53 54] We will calculate the RIS and information size for each outcome. In addition, the cumulative Z-curve's breach relevant to the TSA monitoring boundaries will be quantified for all outcomes.[53 54]

For continuous outcomes, we will calculate the RIS by the observed SD, a mean difference of the observed SD/2 (Difference of SD/2 is considered clinically meaningful), an alpha (type I error) of 2.5% and a beta (type II error) of 10% for primary and secondary outcomes in the

For peer review only - http://bmjopen.bmj.com/site/about/guidelines.xhtml

TSA.[55] For dichotomous outcomes, the proportion of participants with an outcome from the control group, a relative risk reduction/increase of 0.20 (A 20% reduction/increase in relative risk is considered clinically meaningful), and an alpha (type I error) of 2.5% and a beta (type II error) of 0.10 will be used in the TSA.[56] TSA program version 0.9.5.10 beta is available at http://www.ctu.dk/tsa.[57]

The diversity adjusted information size (*DIS*) should be calculated, as the required information size might be underestimated. We will use the formula: DIS= $SS/1- D^2$ (D^2: Diversity, is the percentage of the variability between trials to the within-trial variance and constitutes the percentage of the variability between trials to the total variance in the meta-analysis. *SS*: Sample size in a single randomized clinical trial).[58]

Subgroup analysis

We plan to interpret the results through an analysis of subgroups or subsets. If sufficient trials are available (the subgroup analysis will be performed if the variable is reported by at least 2 RCTs), data from different participants' age, different body mass indexes (BMIs) and different types of MILSs will be analyzed independently.

► Different participants' age (deep NMB for MILS in patients with different ages as follows: 18 years≤ Patients<65 years; 65 years≤ Patients<75 years; Patients≥75 years).

► Different types of MILS (deep NMB for video-assisted thoracoscopic

For peer review only - http://bmjopen.bmj.com/site/about/guidelines.xhtml

lung surgery; deep NMB for robotic-assisted thoracoscopic lung surgery).

► Different BMIs (deep NMB for MILS in patients with different BMIs as follows: BMI<25.0 kg/m^2; 25.0 kg/m^2 ≤ BMI < 30 kg/m^2; BMI ≥ 30kg/m^2).

To determine whether a statistically significant subgroup difference was detected, the p value from the test for subgroup differences will be considered. If a significant difference between subgroups is identified (test for interaction p<0.05), we will report the results for individual subgroups separately. [42]

Sensitivity analysis

After analysis of subgroups or subsets, sensitivity analysis will be applied to evaluate whether the uncertain assumptions of data and usage could affect the stableness of the combined results. We will exclude low-quality studies (defined as high risk of bias studies according to the Cochrane risk of bias tool assessment), then reanalyse the included studies, as to assess whether there are obvious differences between the combined effects. If necessary, we will remove each included study one by one to detect whether the pooled estimations are stable. Significant changes in the combined results may indicate significant heterogeneity among the included studies.

Assessment of publication biases

The potential publication bias will be estimated using the funnel plot

For peer review only - http://bmjopen.bmj.com/site/about/guidelines.xhtml

analysis and Egger's regression test, when more than 10 original studies will be included for an outcome. [59 60] The trim-and-fill analysis will also be applied to confirm any potential publication bias, as it is based on the symmetric pattern of the funnel plot. In the absence of publication bias, the effect sizes of all the studies will be normally distributed around the center of a funnel plot.[61] Stata/MP 16.0 (Stata Corp, College Station, TX, USA) will be applied to perform the publication biases.

Grading the quality of evidence

The quality of evidence for all the outcomes will be assessed using the GRADE approach through risk of bias, consistency, objectivity, accuracy and reported bias. [62] The certainty of evidence will be classified as high, moderate, low or very low. According to GRADE, data from randomized controlled trials are considered high quality evidence but can be rated down according to risk of bias, imprecision, inconsistency, indirectness or publication bias.

Patient and public involvement statement

Patients or the public were not involved in the design, conduct, reporting, or dissemination plans of our research.

DISCUSSION

This systematic review will provide an overview of the current state of evidence on the clinical efficacy of deep NMB for MILS. We will examine the effect of deep NMB on surgical conditions according to the

For peer review only - http://bmjopen.bmj.com/site/about/guidelines.xhtml

surgeon's perspective. In addition, we will evaluate the efficacy of deep NMB on patients' postoperative recovery and postoperative complications. To our knowledge, this will be the first systematic review on this topic. The results of this systematic review will provide evidence for clinical decision making on better management of NMB and patient care during MILS.

This systematic review protocol according to the Preferred Reporting Items for Systematic Review and Meta-Analyses Protocols (PRISMA-P) guidelines. The strengths of our systematic review are as follows: First, we performed a comprehensive search of English and Chinese databases. Second, multivariable analysis (including study quality assessment, subgroup analysis, sensitivity analysis, trial sequential analysis and Egger's regression test) will be performed to control the confounding bias. Third, two independent reviewers will retrieve literature, extract data, and assess study quality according to the guidelines.

Limitations of our systematic review are as follows: First, studies with different NMBA and NMBA antagonists (sugammadex or neostigmine) will be included, leading to potential heterogeneity. Second, the number of studies with available data for subgroup analyses may be limited. Third, the sample size in each included study may be small. Fourth, studies with high-level evidence such as well-designed randomized controlled trials with double-blind designs may be limited. Thus, rigorous meta-analysis

For peer review only - http://bmjopen.bmj.com/site/about/guidelines.xhtml

methods such as TSA and trim-and-fill analysis will be performed in the data analysis, as to confirm the validity of the outcomes. Finally, it is difficult to define a priori a clinical plausible value of relevant mean difference and relative risk increase/decrease during our literature research and clinical experience. Therefore, we defined the clinical plausible value according to the TSA guidelines and the method of sample size calculation.

ETHICS AND DISSEMINATION

Ethical approval was not required for this systematic review protocol. The findings will be disseminated through peer-reviewed publications.

Timelines

Formal screening of search results will begin in March 2022. Data extraction will begin in April 2022. The project will be complete in May 2022.

Author Contributions

Z-JQ and DL conceived the idea for this systematic review. All authors (Z-JQ, DL, WJ, ZL, D-XQ, CG) developed the methodology for the systematic review. The manuscript was drafted by Z-JQ and DL, and revised by all authors. D-XQ and CG will screen potential studies, and perform duplicate independent data abstraction. Z-JQ and ZL will undertake risk of bias assessment and assess the evidence quality. Z-JQ and DL will conduct the data synthesis. All authors contributed to the research and agreed to be responsible for all aspects of the work.

For peer review only - http://bmjopen.bmj.com/site/about/guidelines.xhtml

For peer review only

Funding

None.

Competing interests

None declared.

Data availability statement

Not applicable for this protocol.

Patient consent for publication

No patient was involved.

Provenance and peer review

Not commissioned; externally peer reviewed.

REFERENCES

1 Sung H, Ferlay J, Siegel RL, *et al*. Global Cancer Statistics 2020: GLOBOCAN Estimates of Incidence and Mortality Worldwide for 36 Cancers in 185 Countries. *CA Cancer J Clin* 2021; 71:209-49.

2 McKenna RJ Jr, Houck W, Fuller CB. Video-assisted thoracic surgery lobectomy: experience with 1,100 cases. *Ann Thorac Surg* 2006;81: 421-5; discussion 425-6.

3 Fahim C, Hanna W, Waddell T, *et al*. Robotic-assisted thoracoscopic surgery for lung resection: the first Canadian series. *Can J Surg*2017; 60:260-65.

4 Ujiie H, Gregor A, Yasufuku K. Minimally invasive surgical approaches for lung cancer. *Expert Rev Respir Med* 2019;13(6):571-78.

5 Sihoe AD. Uniportal video-assisted thoracic (VATS) lobectomy. Ann *Cardiothorac Surg* 2016; 5:133-44.

6 Hernandez-Arenas LA, Lin L, Yang Y, *et al*. Initial experience in uniportal subxiphoid video-assisted thoracoscopic surgery for major lung resections. *Eur J Cardiothorac Surg* 2016;50: 1060-66.

7 Ujiie H, Yasufuku K. New era of “resection of the carina and lower trachea. *J Thorac Dis* 2017; 9:4932-36.

8 Ujiie H, Kato T, Hu HP, *et al*. A novel minimally invasive near-infrared thoracoscopic

For peer review only - http://bmjopen.bmj.com/site/about/guidelines.xhtml

localization technique of small pulmonary nodules: A phase I feasibility trial. *J Thorac Cardiovasc Surg* 2017; 154:702-11.

9 Boffa DJ, Kosinski AS, Furnary AP, *et al*. Minimally Invasive Lung Cancer Surgery Performed by Thoracic Surgeons as Effective as Thoracotomy. *J Clin Oncol* 2018; 36:2378-85.

10 Long H, Tan Q, Luo Q, *et al*. Thoracoscopic surgery versus thoracotomy for lung cancer: short-term outcomes of a randomized trial. *Ann Thorac Surg* 2018; 105:386-92.

11 Weksler B, Tavares J, Newhook TE, *et al*. Robot-assisted thymectomy is superior to transsternal thymectomy. *Surg Endosc* 2012; 26:261-6.

12 Han KN, Kim HK, Choi YH. Midterm outcomes of single port thoracoscopic surgery for major pulmonary resection. *PloS one* 2017;12: e0186857.

13 Shen H, Wang X, Nie Y, *et al*. Minimally invasive surgery versus thoracotomy for resectable stage II and III non-small-cell lung cancers: a systematic review and meta-analysis. *Eur J Cardiothorac Surg* 2020; 59: 940-50.

14 O'Sullivan KE, Kreaden US, Hebert AE, *et al*. A systematic review and meta-analysis of robotic versus open and video-assisted thoracoscopic surgery approaches for lobectomy. *Interact Cardiovasc Thorac Surg* 2019; 28:526-34.

15 Yang CJ, Kumar A, Klapper JA, *et al*. A national analysis of long-term survival following thoracoscopic versus open lobectomy for stage I non-small-cell lung cancer. *Annals of surgery* 2019; 269:163-171.

16 O'Sullivan KE, Kreaden US, Hebert AE, *et al*. A systematic review and meta-analysis of robotic versus open and video-assisted thoracoscopic surgery approaches for lobectomy. *Interact Cardiovasc Thorac Surg* 2019; 28:526-34.

17 Li Z, Xia M, Liu C, *et al*. A meta-analysis of minimally invasive surgery versus thoracotomy for centrally located non-small cell lung cancer. *J Thorac Dis* 2021; 13:252-61.

18 Bendixen M, Jørgensen OD, Kronborg C, *et al*. Postoperative pain and quality of life after lobectomy via video-assisted thoracoscopic surgery or anterolateral thoracotomy for early stage lung cancer: a randomised controlled trial. *Lancet Oncol* 2016; 17: 836-

For peer review only - http://bmjopen.bmj.com/site/about/guidelines.xhtml

44.

19 Swanson SJ, Miller DL, McKenna RJ Jr., et al. Comparing robot-assisted thoracic surgical lobectomy with conventional video-assisted thoracic surgical lobectomy and wedge resection: results from a multihospital database (Premier). *J Thorac Cardiovasc Surg* 2014;147: 929-37.

20 Paul S, Jalbert J, Isaacs AJ, et al. Comparative effectiveness of robotic-assisted vs thoracoscopic lobectomy. *Chest* 2014;146: 1505-12.

21 Guerrera F, Olland A, Ruffini E, *et al.* VATS lobectomy vs. open lobectomy for early-stage lung cancer: an endless question-are we close to a definite answer? *J Thorac Dis* 2019; 11:5616-8.

22 Swanson SJ, Herndon JE, D'Amico TA, et al. Video- assisted thoracic surgery lobectomy: report of CALGB 39802-a prospective, multi-institution feasibility study. *J Clin Oncol* 2007; 25:4993-7.

23 Cao C, Manganas C, Ang SC, *et al*. Video-assisted thoracic surgery versus open thoracotomy for non-small cell lung cancer: a meta-analysis of propensity score-matched patients. *Interact Cardiovasc Thorac Surg* 2013; 16:244-9.

24 Guerrera F, Olland A, Ruffini E, *et al*. VATS lobectomy vs. open lobectomy for early-stage lung cancer: an endless question-are we close to a definite answer? *J Thorac Dis* 2019;11: 5616-8.

25 Handy JR Jr. Minimally invasive lung surgery and postoperative quality of life. *Thorac Surg Clin* 2012;22:487-95.

26 Iwata H. Minimally invasive pulmonary surgery for lung cancer, up to date. *Gen Thorac Cardiovasc Surg* 2013; 61:449–54.

27 Barrio J, Errando CL, Miguel GS, *et al*. Effect of depth of neuromuscular blockade on the abdominal space during pneumoperitoneum establishment in laparoscopic surgery. *J Clin Anesth* 2016; 34:197-203.

28 Kim HJ, Lee K, Park WK, *et al*. Deep neuromuscular block improves the surgical conditions for laryngeal microsurgery. *Br J Anaesth* 2015; 115:867-72.

29 Lesser TG, Schubert H, Güllmar D, *et al*. One-lung flooding reduces the ipsilateral diaphragm motion during mechanical ventilation. *Eur J Med Res* 2016; 21:9.

For peer review only - http://bmjopen.bmj.com/site/about/guidelines.xhtml

30 Gibson PG, Simpson JL, Ryan NM, *et al*. Mechanisms of cough. *Curr Opin Allergy Clin Immunol* 2014; 14:55-61.

31 Fernando PU, Viby-Mogensen J, Bonsu AK, *et al*. Relationship between posttetanic count and response to carinal stimulation during vecuronium-induced neuromuscular blockade. *Acta Anaesthesiol Scand* 1987;31: 593-6.

32 Dhonneur G, Kirov K, Motamed C, *et al.* Post-tetanic count at adductor pollicis is a better indicator of early diaphragmatic recovery than train-of-four count at corrugator supercilii. *Br J Anaesth* 2007;99: 376–9.

33 Casanova J, Piñeiro P, De La Gala F, *et al*. Deep versus moderate neuromuscular block during one-lung ventilation in lung resection surgery. *Rev Bras Anestesiol* 2017;67: 288-93.

34 Fortier LP, McKeen D, Turner K, *et al.* The RECITE study: a Canadian Prospective, Multicenter Study of the incidence and severity of residual neuromuscular blockade. *Anesth Analg* 2015; 121:366-72.

35 Raval AD, Uyei J, Karabis A, *et al.* Incidence of residual neuromuscular blockade and use of neuromuscular blocking agents with or without antagonists: A systematic review and meta-analysis of randomized controlled trials. *J Clin Anesth* 2020; 64:109818.

36 Kheterpal S, Vaughn MT, Dubovoy TZ, *et al*. Sugammadex versus Neostigmine for Reversal of Neuromuscular Blockade and Postoperative Pulmonary Complications (STRONGER): A Multicenter Matched Cohort Analysis. *Anesthesiology* 2020; 132:1371-81.

37 Cammu G. Residual Neuromuscular Blockade and Postoperative Pulmonary Complications: What Does the Recent Evidence Demonstrate? *Curr Anesthesiol Rep* 2020; 27:1-6.

38 Grabitz SD, Rajaratnam N, Chhagani K, *et al*. The Effects of Postoperative Residual Neuromuscular Blockade on Hospital Costs and Intensive Care Unit Admission: A Population-Based Cohort Study. *Anesth Analg* 2019; 128:1129-36.

39 Stawicki N, Gessner P. Residual Neuromuscular Blockade in the Critical Care Setting. *AACN Adv Crit Care*2018;29: 15-24.

40 Saager L, Maiese EM, Bash LD, *et al*. Incidence, risk factors, and consequences of

For peer review only - http://bmjopen.bmj.com/site/about/guidelines.xhtml

residual neuromuscular block in the United States: The prospective, observational, multicenter RECITE-US study. *J Clin Anesth* 2019; 55:33-41.

41 Shamseer L, Moher D, Clarke M. Preferred reporting items for systematic review and meta-analysis protocols (PRISMA-P) 2015: elaboration and explanation. *BMJ* 2015; 350: g7647.

42 Higgins JPT, Thomas J, Chandler J, *et al*. Cochrane Handbook for Systematic Reviews of Interventions version 6.2 (updated February 2021). Cochrane, 2021. Available: www.training.cochrane.org/handbook.

43 Page MJ, McKenzie JE, Bossuyt PM, *et al*. The PRISMA 2020 statement: an updated guideline for reporting systematic reviews. *BMJ* 2021;372: n71.

44 Brull SJ, Kopman AF. Current Status of Neuromuscular Reversal and Monitoring: Challenges and Opportunities. Anesthesiology. 2017 Jan;126(1):173-190.

45 Martini CH, Boon M, Bevers RF, *et al*. Evaluation of surgical conditions during laparoscopic surgery in patients with moderate vs. deep neuromuscular block. *Br J Anaesth* 2014;112: 498-505.

46 Gheibi S, Mahmoodzadeh A, Kashfi K, *et al*. Data Extraction from Graphs Using Adobe Photoshop: Applications for Meta-Analyses. *Int J Endocrinol Metab* 2019;17: e95216.

47 Higgins JPT, Altman DG, Gøtzsche PC, *et al*. The Cochrane collaboration's tool for assessing risk of bias in randomised trials. *BMJ* 2011; 343: d5928.

48 Higgins JPT, Savović J, Page MJ, et al. Chapter 8: Assessing risk of bias in a randomized trial. In: Higgins JPT, Thomas J, Chandler J, Cumpston M, Li T, Page MJ, Welch VA (editors). Cochrane Handbook for Systematic Reviews of Interventions version 6.2 (updated February 2021). Cochrane, 2021. Available from www.training.cochrane.org/handbook.

49 Savović J, Turner RM, Mawdsley D, *et al*. Association between Risk of-Bias assessments and results of randomized trials in Cochrane reviews: the ROBES Meta-Epidemiologic study. *Am J Epidemiol* 2018;187: 1113-22.

50 Wetterslev J, Thorlund K, Brok J, *et al*. Estimating required information size by quantifying diversity in random-effects model meta-analyses. *BMC Med Res Methodol*

For peer review only

2009; 9:86.

51 Wetterslev J, Jakobsen JC, Gluud C. Trial sequential analysis in systematic reviews with meta-analysis. *BMC Med Res Methodol* 2017; 17:39.

52 Wetterslev J, Thorlund K, Brok J, *et al*. Trial sequential analysis may establish when firm evidence is reached in cumulative meta-analysis. *J Clin Epidemiol* 2008; 61: 64-75.

53 Imberger G, Thorlund K, Gluud C, *et al*. False-Positive findings in Cochrane meta-analyses with and without application of trial sequential analysis: an empirical review. *BMJ Open* 2016;6: e011890.

54 Brok J, Thorlund K, Gluud C, *et al*. Trial sequential analysis reveals insufficient information size and potentially false positive results in many meta-analyses. *J Clin Epidemiol* 2008; 61:763-9.

55 Norman GR, Sloan JA, Wyrwich KW. Interpretation of changes in health-related quality of life: the remarkable universality of half a standard deviation. *Med Care* 2003; 41:582-92.

56 Castellini G, Nielsen EE, Gluud C. Comment on: "Cell therapy for heart disease: Trial sequential analyses of two cochrane reviews". *Clin Pharmacol Ther* 2017; 102:21-4.

57 Thorlund K, Engstrøm J, Wetterslev J, *et al.* User manual for trial sequential analysis (TSA) Copenhagen trial unit, centre for clinical intervention research, Denmark; 2011.Available: http://www.ctu.dk/tsa [Accessed 2021].

58 Wetterslev J, Thorlund K, Brok J, *et al.* Estimating required information size by quantifying diversity in random-effects model meta-analyses. *BMC Med Res Methodol* 2009; 9:86.

59 Egger M, Davey Smith G, Schneider M, *et al*. Bias in meta-analysis detected by a simple, graphical test. *BMJ* 1997;315: 629-34.

60 Sterne JA, Gavaghan D, Egger M. Publication and related bias in meta-analysis: power of statistical tests and prevalence in the literature. *J Clin Epidemiol* 2000; 53:1119-29.

61 Mavridis D, Salanti G. How to assess publication bias: funnel plot, trim-and-fill method and selection models. *Evid Based Ment Health* 2014; 17:30.

For peer review only - http://bmjopen.bmj.com/site/about/guidelines.xhtml

62 Guyatt G, Oxman AD, Akl EA, *et al.* Grade guidelines: 1. Introduction-GRADE evidence profles and summary of fndings tables. *J Clin Epidemiol* 2011; 64:383-94.

Figure Legends

Figure 1. The PRISMA flow diagram.

For peer review only

For peer review only - http://bmjopen.bmj.com/site/about/guidelines.xhtml

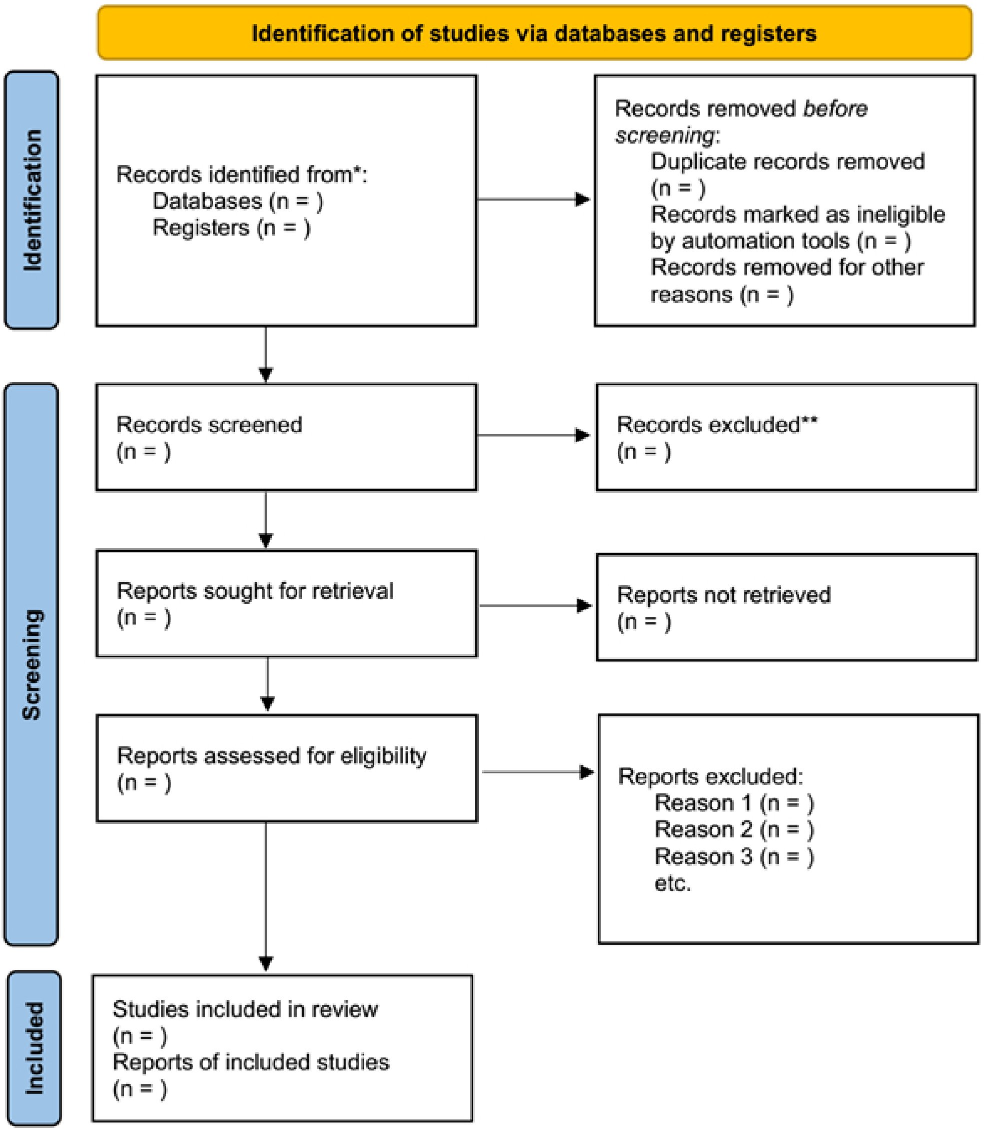

Figure 1 PRISMA_2020_flow_diagram

For peer review only - http://bmjopen.bmj.com/site/about/guidelines.xhtml

Supplementary appendix 1: Search strategy

Search strategy of PubMed as follows:

#1 “neuromuscular blockade"[MeSH Terms] OR neuromusc*[tiab]” OR “muscle relaxation” [MeSH Terms]

#2 “Deep[tiab] OR profound[tiab] OR intense[tiab] OR extreme[tiab] OR depth[tiab]”

#3 “Pulmonary” [Mesh] OR “Lung” [Mesh] OR Pulmonary [tiab] OR Lung [tiab]

#4 “Surgical Procedures Operative” [Mesh] OR “Microsurgery” [Mesh] OR “Surgical Procedures Minimally Invasive” [Mesh] OR Minimally Invasive Surgery[tiab] OR MIS [tiab] OR Minimal Access Surgical Procedures [tiab]OR Minimal Surgical Procedures[tiab] OR Minimally Invasive Surgical Procedures [tiab] OR Minimal Surgical Procedure[tiab] OR minimally invasive surgical procedure [tiab] OR minimal access surgical procedure[tiab]

#5 “Thoracic surgery, Video-Assisted” [Mesh] or Surgeries, Video-Assisted Thoracic [af] or Surgery, Video-Assisted Thoracic [af] or Thoracic Surgeries, Video-Assisted [af] or Thoracic surgery, Video-Assisted [af] or Video-Assisted Thoracic Surgeries [af] or Video-Assisted Thoracic Surgery [af] or Surgeries, Video-Assisted Thoracoscopic [af] or Surgery, Video-Assisted Thoracoscopic [af] or Thoracoscopic Surgeries, Video-Assisted [af] or Thoracoscopic Surgery, Video-Assisted [af] or Video Assisted Thoracoscopic Surgery [af] or Video Assisted Thoracoscopic Surgeries [af] or Video-Assisted Thoracic Surgery [af] or Video Assisted Thoracic Surgery [af] or Surgery, Thoracic, Video-Assisted [af] or VATS [af] or VATSs [af].

#6 “Robotics” [MeSH] OR robot* [tiab] OR computer guid*[tiab] OR computer-guid*[tiab] OR computer-assisted[tiab] OR computer assisted [tiab]OR da Vinci [tiab]OR Zeus [tiab]OR telesurgery[tiab]

#7 #1 AND #2 AND #3

#8 #4 OR #5 OR #6

#9 “controlled clinical trial” [Publication Type] OR “randomized controlled trial” [Publication Type] OR “randomized” [Title/Abstract] OR “randomized” [Title/Abstract] OR “Placebo” [Title/Abstract] OR “randomly” [Title/Abstract] OR “Clinical trial” [Title]

#10 (animals [MeSH Terms]) NOT ((human [MeSH Terms]) AND (animals [MeSH Terms]))

#11 #7and #8 and #9 not #10

Search strategy of Cochrane library as follows:

For peer review only

#1 MeSH descriptor: [neuromuscular blockade] explode all trees

#2 MeSH descriptor: [muscle relaxation] explode all trees

#3 (neuromusc*): ti,ab,kw

#4 #1 or # 2 or # 3

#5 (Deep): ti,ab,kw or (profound):ti,ab,kw or (intense):ti,ab,kw or (extreme):ti, ab,kw or (depth):ti,ab,kw

#6 #4 and #5

#7 MeSH descriptor: [Pulmonary] explode all trees

#8 MeSH descriptor: [Lung] explode all trees

#9 #7 or # 8

#10 #6 and #9

#11 MeSH descriptor: [Surgical Procedures Operative] explode all trees

#12 MeSH descriptor: [Thoracic surgery, Video-Assisted] explode all trees

#13 MeSH descriptor: [Microsurgery] explode all trees

#14 MeSH descriptor: [Surgical Procedures Minimally Invasive] explode all trees

#15 MeSH descriptor: [Robotics] explode all trees

#16 (surgery or surgical* or Video-Assisted* or Video Assisted* or Video* or Thorac* or VATS): ti,ab,kw

#17 (robot*): ti,ab,kw

#18 (computer guid* OR computer-guid* OR computer-assisted OR computer assisted): ti,ab,kw

#19 (da Vinci OR Zeus OR telesurgery): ti,ab,kw

#20 #11 or # 12 or #13 or # 14 or #15 or # 16 or #17 or # 18 or #19

#21 #10 and # 20

#22 (controlled clinical trial):pt or (randomized controlled trial):pt or (random*): ti,ab,kw or (Clinical trial):ti,ab,kw

#23 #21 and #22

Search strategy of Web of Science as follows:

#1 TS= (neuromuscular blockade or neuromusc* or muscle relaxation)

#2 TS= (Deep or profound or intense or extreme or depth)

#3 TS= (Pulmonary or Lung)

For peer review only

#4 TS= (Surgical Procedures Operative or Thoracic surgery, Video-Assisted or Microsurgery or Surgical Procedures Minimally Invasive or Robotics)

#5TS= (surgery or surgical* or Video-Assisted* or Video Assisted* or Video* or Thorac* or VATS or robot* or computer guid* or computer-guid* or computer-assisted or computer assisted or da Vinci or Zeus or telesurgery)

#6 #4 OR #5

#7 #1 and #2 and #3 and #6

#8 TS= (random* or Clinical trial)

#9 #7 and #8

Search strategy for Ovid Medline as follows:

#1 exp neuromuscular blockade/ or exp muscle relaxation/ or neuromusc*.mp.

#2 (Deep or profound or intense or extreme or depth) .mp.

#3 exp pulmonary / or exp Lung

#4 #1 and #2 and #3

#5 exp surgical procedures operative/ or exp Thoracic surgery, Video-Assisted / or exp Microsurgery/or exp Surgical Procedures Minimally Invasive/ or exp Robotics/

#6 (surgery or surgical*).mp.

#7 (Video-Assisted* or Video Assisted* or Video* or Thorac* or VATS).mp.

#8 (robot*).mp.

#9(computer guid* or computer-guid* or computer-assisted or computer assisted).mp.

#10(da Vinci or Zeus or telesurgery).mp.

#11 #5or #6 or #7 or #8 or #9 or #10

#12 randomized controlled trial.pt.

#13 controlled clinical trial.pt.

#14 randomized.ab.

#15 placebo.ab.

#16 clinical trials as topic.sh.

#17 randomly.ab.

#18 trial.ti.

#19 #12 or #13 or #14 or #15 or #16 or #17 or #18

For peer review only

#20 (animals not (humans and animals)).sh.

#21 #19 not #20

#22 #4 and #11 and #21

Search strategy for Embase as follows:

#1 exp neuromuscular blockade/

#2 neuromusc*.mp.

#3 exp muscle relaxation/

#4 (Deep or profound or intense or extreme or depth).mp.

#5 exp Pulmonary /

#6 exp Lung/

#7 exp Surgical Procedures Operative/

#8 exp Thoracic surgery, Video-Assisted /

#9 exp Microsurgery/

#10 exp Surgical Procedures Minimally Invasive/

#11 exp Robotic/

#12 (surgery or surgical*).mp.

#13 (robot*).mp.

#14(Video-Assisted* or Video Assisted* or Video* or Thorac* or VATS).mp.

#15 (computer guid* or computer-guid* or computer-assisted or computer assisted).mp.

#16 (da Vinci or Zeus or telesurgery).mp.

#17 Clinical trial.mp.

#18 (placebo*).mp.

#19 exp randomized controlled trial/

#20 (random*).mp.

#21 (exp animal/ or nonhuman/ or exp animal experiment/) not human/

#22 #1 or #2 or #3

#23 #22 and #4

#24 #5 or #6

#25 #7 or #8 or #9 or #10 or #11 or #12 or #13 or #14 or #15 or #16

#26 #23 and #24 and #25

For peer review only

#27 #17 or #18 or #19 or #20

#28 #26 and #27

#29 #28 not #21

WHO ICTRP Trial registry

http://apps.who.int/trialsearch (WHO ICTRP register) will be searched via the advanced search page. Search terms were: (Lung or Pulmonary) AND (minimal invasive or minimally invasive or surgery or surgical procedures operative or microsurgery or Video-Assisted* or Video Assisted* or Video* or Thorac* or VATS or robot* or computer guid* or computer-guid* or computer-assisted or computer assisted or da Vinci or Zeus or telesurgery) AND (Deep neuromuscular blockade or profound neuromuscular blockade or intense neuromuscular blockade or extreme neuromuscular blockade or depth neuromuscular blockade).

Clinicaltrials.gov search strategy

http://clinicaltrials.gov (NIH register) will be searched via advanced search page. Search terms were: Condition or disease: (Lung or Pulmonary) AND (minimal invasive or minimally invasive or surgery or surgical procedures operative or microsurgery or Video-Assisted* or Video Assisted* or Video* or Thorac* or VATS or robot* or computer guid* or computer-guid* or computer-assisted or computer assisted or da Vinci or Zeus or telesurgery).

Study type: Interventional Studies.

Intervention/treatment: (Deep neuromuscular blockade or profound neuromuscular blockade or intense neuromuscular blockade or extreme neuromuscular blockade or depth neuromuscular blockade)

Chinese database

China National Knowledge Infrastructure (CNKI) search strategy

(电视胸腔镜 or 胸腔镜 or 腔镜 or 微创 or 机器人 or 机器人辅助 or 达芬奇 or 宙斯 or RATS or VATS or 多孔 VATS or 四孔 VATS or 三孔 VATS or 两孔 VATS or 单孔 VATS) and (手术 or 切除术 or 根治术) and (肺 or 肺癌 or 肺肿瘤) and (深度肌松 or 深肌松 or 深度神经肌肉阻滞)and (随机 or 对照)

Chinese BioMedical Literature (CBM)

("电视胸腔镜" [全部字段] or"胸腔镜" [全部字段] or "腔镜" [全部字段] or "微创" [全部字段]

For peer review only - http://bmjopen.bmj.com/site/about/guidelines.xhtml

or "机器人" [全部字段] or "机器人辅助" [全部字段] or "达芬奇" [全部字段] or "宙斯" [全部字段] or " RATS " [全部字段] or "VATS [全部字段] or "多孔 VATS" [全部字段]or "四孔 VATS " [全部字段] or "三孔 VATS" [全部字段] or "两孔 VATS" [全部字段] or "单孔 VATS" [全部字段]) and ("手术" [全部字段] or "切除术" [全部字段] or"根治术" [全部字段]) and ("肺" [全部字段] or "肺癌" [全部字段] or"肺肿瘤" [全部字段]) and ("深度肌松" [全部字段] or "深肌松" [全部字段] or"深度神经肌肉阻滞" [全部字段]) and ("随机" [全部字段] or "对照" [全部字段])

VIP database

关键词=(电视胸腔镜 or 胸腔镜 or 腔镜 or 微创 or 机器人 or 机器人辅助 or 达芬奇 or 宙斯 or RATS or VATS or 多孔 VATS or 四孔 VATS or 三孔 VATS or 两孔 VATS or 单孔 VATS) AND 关键词= (手术 or 切除术 or 根治术) AND 关键词= (肺 or 肺癌 or 肺肿瘤) AND 关键词= (深度肌松 or 深肌松 or 深度神经肌肉阻滞)AND 关键词= (随机 or 对照)

Wan fang database.

(电视胸腔镜 or 胸腔镜 or 腔镜 or 微创 or 机器人 or 机器人辅助 or 达芬奇 or 宙斯 or RATS or VATS or 多孔 VATS or 四孔 VATS or 三孔 VATS or 两孔 VATS or 单孔 VATS) and (手术 or 切除术 or 根治术) and (肺 or 肺癌 or 肺肿瘤) and (深度肌松 or 深肌松 or 深度神经肌肉阻滞)and (随机 or 对照)

For peer review only

Assessment of risk of bias in included studies

Random sequence generation

- ***Low risk:*** If sequence generation was achieved using computer random number generator or a random number table. Drawing lots, tossing a coin, shuffling cards, and throwing dice were also considered adequate if performed by an independent adjudicator.
- ***Unclear risk:*** If the method of randomisation was not specified, but the trial was still presented as being randomised.
- ***High risk:*** If the allocation sequence is not randomised or only quasi-randomised. These trials will be excluded.

Allocation concealment

- ***Low risk:*** If the allocation of patients was performed by a central independent unit, onsite locked computer or identical-looking numbered sealed envelopes.
- ***Uncertain risk:*** If the trial was classified as randomised but the allocation concealment process was not described.
- ***High risk:*** If the allocation sequence was familiar to the investigators who assigned participants.

Blinding of participants and treatment providers

- ***Low risk:*** If the participants and the treatment providers were blinded to intervention allocation and this was described.
- ***Uncertain risk:*** If the procedure of blinding was insufficiently described.
- ***High risk:*** If blinding of participants and the treatment providers was not performed.

Blinding of outcome assessment

- ***Low risk of bias:*** If it was mentioned that outcome assessors were blinded and this was described.
- ***Uncertain risk of bias:*** If it was not mentioned if the outcome assessors in the trial were blinded or the extent of blinding was insufficiently described.
- ***High risk of bias:*** If no blinding or incomplete blinding of outcome assessors was performed.

Incomplete outcome data

- ***Low risk of bias:*** If missing data were unlikely to make treatment effects depart from plausible

For peer review only

values. This could be either (1) there were no drop-outs or withdrawals for all outcomes, or (2) the numbers and reasons for the withdrawals and drop-outs for all outcomes were clearly stated and could be described as being similar to both groups. Generally, the trial is judged as at a low risk of bias due to incomplete outcome data if drop-outs are less than 5%. However, the 5% cut-off is not definitive.

- ***Uncertain risk of bias:*** If there was insufficient information to assess whether missing data were likely to induce bias on the results.
- ***High risk of bias:*** If the results were likely to be biased due to missing data either because the pattern of drop-outs could be described as being different in the two intervention groups or the trial used improper methods in dealing with the missing data (e.g. last observation carried forward).

Selective outcome reporting

- ***Low risk of bias:*** If a protocol was published before or at the time the trial was begun and the outcomes specified in the protocol were reported on. If there is no protocol or the protocol was published after the trial has begun, reporting of serious adverse events will grant the trial a grade of low risk of bias.
- ***Uncertain risk of bias:*** If no protocol was published and the outcome of serious adverse events were not reported on.
- ***High risk of bias:*** If the outcomes in the protocol were not reported on.

Other risks of bias

- ***Low risk of bias:*** If the trial appears to be free of other components that could put it at risk of bias.
- ***Unclear risk of bias:*** If the trial may or may not be free of other components that could put it at risk of bias.
- ***High risk of bias:*** If there are other factors in the trial that could put it at risk of bias (including, Design-specific risk of bias, stopped early due to some data-dependent process including a formal-stopping rule, baseline imbalance, claimed fraudulent, blocked randomization in unblinded trials, differential diagnostic activity, contamination, inappropriate measurement instrument for outcomes, deviation from the study protocol unrelated to the clinical practice, authors conducted trials on the same topic, academic bias, for-profit bias, inappropriate

For peer review only

financial conflict of interest).

Overall risk of bias

- ***Low risk of bias:*** The trial will be classified as overall 'low risk of bias' only if all of the bias domains described in the above paragraphs are classified as 'low risk of bias'.
- ***High risk of bias:*** The trial will be classified as 'high risk of bias' if any of the bias risk domains described in the above are classified as 'unclear' or 'high risk of bias'.
- We will assess the domains 'blinding of outcome assessment', 'incomplete outcome data', and 'selective out- come reporting' for each outcome result. Thus, we can assess the bias risk for each outcome assessed in addition to each trial. Our primary conclusions will be based on the results of our primary outcome results with overall low risk of bias. Both our primary and secondary conclusions will be presented in the summary of findings tables.

For peer review only

PRISMA-P checklist

Table PRISMA-P (preferred reporting items for systematic review and meta-analysis protocols) 2015 checklist: recommended items to address in a systematic review protocol

Section and topic	Item No	Checklist item	Reported on page #
**Administrative information**			
Title:			
Identification	1a	Identify the report as a protocol of a systematic review	1
Update	1b	If the protocol is for an update of a previous systematic review, identify as such	None
Registration	2	If registered, provide the name of the registry (such as PROSPERO) and registration number	3,6
Authors:			
Contact	3a	Provide name, institutional affiliation, e-mail address of all protocol authors; provide physical mailing address of corresponding author	1
Contributions	3b	Describe contributions of protocol authors and identify the guarantor of the review	19
Amendments	4	If the protocol represents an amendment of a previously completed or published protocol, identify as such and list changes; otherwise, state plan for documenting important protocol amendments	None
Support:			
Sponsor	5b	Provide name for the review funder and/or sponsor	None
Role of sponsor or funder	5c	Describe roles of funder(s), sponsor(s), and/or institution(s), if any, in developing the protocol	None
**Introduction**			
Rationale	6	Describe the rationale for the review in the context of what is already known	3-6
Objectives	7	Provide an explicit statement of the question(s) the review will address with reference to participants, interventions, comparators, and outcomes (PICO)	6-8
**Methods**			
Eligibility criteria	8	Specify the study characteristics (such as PICO, study design, setting, time frame) and report characteristics (such as years considered, language, publication status) to be used as criteria for eligibility for the review	6-9

Information sources	9	Describe all intended information sources (such as electronic databases, contact with study authors, trial registers or other grey literature sources) with planned dates of coverage	9-11
Search strategy	10	Present draft of search strategy to be used for at least one electronic database, including planned limits, such that it could be repeated	10-11, S1
Study records:			
Data management	11a	Describe the mechanism(s) that will be used to manage records and data throughout the review	11
Selection process	11b	State the process that will be used for selecting studies (such as two independent reviewers) through each phase of the review (that is, screening, eligibility and inclusion in meta-analysis)	12-13
Data collection process	11c	Describe planned method of extracting data from reports (such as piloting forms, done independently, in duplicate), any processes for obtaining and confirming data from investigators	12-13
Data items	12	List and define all variables for which data will be sought (such as PICO items, funding sources), any pre-planned data assumptions and simplifications	11-12
Outcomes and prioritization	13	List and define all outcomes for which data will be sought, including prioritization of main and additional outcomes, with rationale	8-9
Risk of bias in individual studies	14	Describe anticipated methods for assessing risk of bias of individual studies, including whether this will be done at the outcome or study level, or both; state how this information will be used in data synthesis	13-16
Data synthesis	15a	Describe criteria under which study data will be quantitatively synthesised	15-16
	15b	If data are appropriate for quantitative synthesis, describe planned summary measures, methods of handling data and methods of combining data from studies, including any planned exploration of consistency (such as I^2, Kendall's τ)	16
	15c	Describe any proposed additional analyses (such as sensitivity or subgroup analyses, meta-regression)	15-17
	15d	If quantitative synthesis is not appropriate, describe the type of summary planned	14-15
Meta-bias(es)	16	Specify any planned assessment of meta-bias(es) (such as publication bias across studies, selective reporting within studies)	18
Confidence in cumulative evidence	17	Describe how the strength of the body of evidence will be assessed (such as GRADE)	14-17
